# GRAM: A GeneRAlized Model to predict the molecular effect of a non-coding variant in a cell-type specific manner

**DOI:** 10.1371/journal.pgen.1007860

**Published:** 2019-08-30

**Authors:** Shaoke Lou, Kellie A. Cotter, Tianxiao Li, Jin Liang, Hussein Mohsen, Jason Liu, Jing Zhang, Sandra Cohen, Jinrui Xu, Haiyuan Yu, Mark A. Rubin, Mark Gerstein

**Affiliations:** 1 Program in Computational Biology and Bioinformatics, Yale University, New Haven, Connecticut, United States of America; 2 Department of Molecular Biophysics and Biochemistry, Yale University, New Haven, Connecticut, United States of America; 3 Department for BioMedical Research, University of Bern, CH, Bern, Switzerland; 4 Weill Institute for Cell and Molecular Biology, Cornell University, Ithaca, New York, United States of America; 5 Program in the History of Science and Medicine, Yale University, New Haven, Connecticut, United States of America; 6 Department of Pathology and Laboratory Medicine, Weill Cornell Medicine, Cornell University, New York, New York, United States of America; 7 Department of Computational Biology, Cornell University, Ithaca, New York, United States of America; 8 Weill Cornell Medicine, New York, United States of America; Stanford University, UNITED STATES

## Abstract

There has been much effort to prioritize genomic variants with respect to their impact on “function”. However, function is often not precisely defined: sometimes it is the disease association of a variant; on other occasions, it reflects a molecular effect on transcription or epigenetics. Here, we coupled multiple genomic predictors to build GRAM, a *G*ene*RA*lized *M*odel, to predict a well-defined experimental target: the expression-modulating effect of a non-coding variant on its associated gene, in a transferable, cell-specific manner. Firstly, we performed feature engineering: using LASSO, a regularized linear model, we found transcription factor (TF) binding most predictive, especially for TFs that are hubs in the regulatory network; in contrast, evolutionary conservation, a popular feature in many other variant-impact predictors, has almost no contribution. Moreover, TF binding inferred from *in vitro* SELEX is as effective as that from *in vivo* ChIP-Seq. Second, we implemented GRAM integrating only SELEX features and expression profiles; thus, the program combines a universal regulatory score with an easily obtainable modifier reflecting the particular cell type. We benchmarked GRAM on large-scale MPRA datasets, achieving AUROC scores of 0.72 in GM12878 and 0.66 in a multi-cell line dataset. We then evaluated the performance of GRAM on targeted regions using luciferase assays in the MCF7 and K562 cell lines. We noted that changing the insertion position of the construct relative to the reporter gene gave very different results, highlighting the importance of carefully defining the exact prediction target of the model. Finally, we illustrated the utility of GRAM in fine-mapping causal variants and developed a practical software pipeline to carry this out. In particular, we demonstrated in specific examples how the pipeline could pinpoint variants that directly modulate gene expression within a larger linkage-disequilibrium block associated with a phenotype of interest (e.g., for an eQTL).

## Author summary

With advances in sequencing technologies, a deluge of genomic data is available; however, only a fraction of non-coding genomic variants are functionally relevant. Sifting through this data to prioritize genomic variants with respect to function is an important but challenging task. In this study, we built GRAM, a GeneRAlized Model, to predict the expression-modulating effects of non-coding variants in a cell-specific manner. GRAM combines a universal regulatory score defined by transcription factor binding with an easily obtainable modifier defined by transcription factor binding and expression to reflect the particular cell type. We evaluated this framework on multiple cell lines with high performance and showed that it could be applied to any cell line or sample with gene expression data. We also integrated GRAM into a practical software pipeline to fine-map causal variants that directly modulate gene expression among a larger linkage-disequilibrium block associated with a phenotype of interest. GRAM complements other general variant effect prediction methods–which often combine disparate features–by helping to precisely define the subset of prioritized variants that directly alters gene expression.

## Introduction

Advances in next-generation sequencing (NGS) technologies have enabled high-throughput whole genome and exome sequencing [1], which have led to the identification and characterization of many disease-associated mutations [2] and the vast majority of common single nucleotide variants (SNVs) in the human population [3, 4]. Genome-wide association studies (GWAS) have found that these variants mostly lie outside of protein-coding regions [5], emphasizing the functional importance of non-coding regulatory elements in the human genome. These advances have also led to an urgent need to develop high-throughput methods to sift through this deluge of sequencing data to quickly determine the functional relevance of each non-coding variant [6].

Evidence suggests that only a fraction of non-coding variants are functional, and the majority of functional variants show only modest effects [7]. Studies like GWAS [8] and expression quantitative trait eQTL [9] have evaluated the association of variants with traits of interest from a statistical perspective. In traditional GWAS and eQTL analyses, an association locus may host the tag-SNPs and a number of linked variants that may potentially account for the molecular mechanism underlying the association [10]. However, it remains difficult to distinguish those that are truly causal [11–13]. Thus, downstream analysis requires fine-mapping to identify the true causal variants by integrating the external genetic and epigenetic information [12, 14].

As association studies give little information about the mechanism of a variant’s effects, it would be helpful to directly test the molecular effects of a large numbers of variants using highly quantitative assays. Luciferase reporter assays are a common method to measure the regulatory effects of functional elements [15]. Researchers can compare the difference of luciferase expression with and without a mutation to estimate the experimental molecular effect of non-coding variants lying in a functional element. By using high-throughput microarray and NGS technology, the massively parallel reporter assay (MPRA) has extended the scales to the genome-wide level [16–21]. Recently, Tewhey and colleagues demonstrated the capability of MPRA to identify the causal variants that directly modulated gene expression [22, 23]. This study identified 842 expression-modulating variants (emVARs) showing significantly differential expression modulation effects and provided a high-quality data source for computational modeling [22, 23].

There is an increasing need for computational methods to effectively predict the molecular effects of variants and improve our understanding of the underlying biology of these effects. Several approaches have been developed to address the problem of variant prioritization from different perspectives. Based on the target of predictions, these methods roughly fall into three major categories: 1) disease-causing effect predictors (e.g. GWAVA [24], and GenoSkyline [25]), which aim to prioritize causal disease variants and distinguish them from benign ones; 2) fitness consequence prioritization tools (e.g., CADD [26], fitCons [27] and LINSIGHT [28]), which attempt to identify the variants based on evolutionary fitness; 3) comprehensive tools (e.g., DeepSEA [29], FunSeq2 [6], FUN-LDA [30]) which integrate multiple data sources for prediction of functional variants. Many of these computational methods are designed to predict and prioritize deleterious and disease-associated variants from a phenotypic perspective, but not to highlight specific molecular consequences of these variants (i.e., their effects on the activities of functional elements). Moreover, some of these tools are cell type-agnostic, and tools that are cell type-aware depend on cell type-specific data with somewhat limited availability, such as ChIP-Seq or epigenetic features. Thus, it would be helpful to build a generalized model that can be systematically specialized to any desired cell type with only a small amount of easily obtainable cell type-specific information (e.g. expression data).

In this study, we addressed the problem of molecular effect prediction of variants from a different perspective. Instead of predicting phenotypic consequences from genotypes, which is a common practice, we aimed to directly predict the expression-modulating effect of the variants from various sources of information. Our model, named GRAM (i.e., GeneRAlized Model), incorporates selected transcription factor (TF) binding information from in vitro SELEX assays, representing the general binding affinity of TFs on the variant’s location, and cell type-specific expression profiles, representing cellular contexts. Combining cell type-independent and -dependent features makes our model both flexible and specific. When we evaluated results from MPRA and luciferase assay experiments show our model achieved high predictive performance and could be easily transferred to other cell types and assay platforms. We also demonstrated the potential application of GRAM to the fine-mapping of pre-defined variants in linkage disequilibrium. As a supplement to many general variant effect prediction methods (which often combine disparate features), our model can help to precisely define the subset of prioritized variants that directly alters gene expression. For instance, after using a more general functional impact tool such as FunSeq or VEP [31, 32], one could use GRAM on the prioritized variants to identify the subset that has a direct expression modulating effect (as opposed to being prioritized for other reasons such as strong association with an organismal phenotype). Furthermore, one could use GRAM to fine-map the key causal variant modulating gene expression from the many variants in a linkage-disequilibrium block associated with gene expression in an eQTL study.

## Results

### Overall analysis flow

In this study, we first collected a dataset from Tewhey et al. [22] to estimate expression modulation differences between reference allele and mutants in the GM12878 cell line. This MPRA-generated dataset contains 3,222 SNVs filtered by logSkew value, which measures the log-fold change of the expression-modulating differences between reference and alternative alleles. Among them, 792 variants (named emVARs) had a significant expression-modulating effect compared with their respective reference allele, which indicates the molecular effect of the variant. Here, we treated emVARs and non-emVARs as positive and negative dataset, respectively, in our GRAM model.

**Fig 1:**
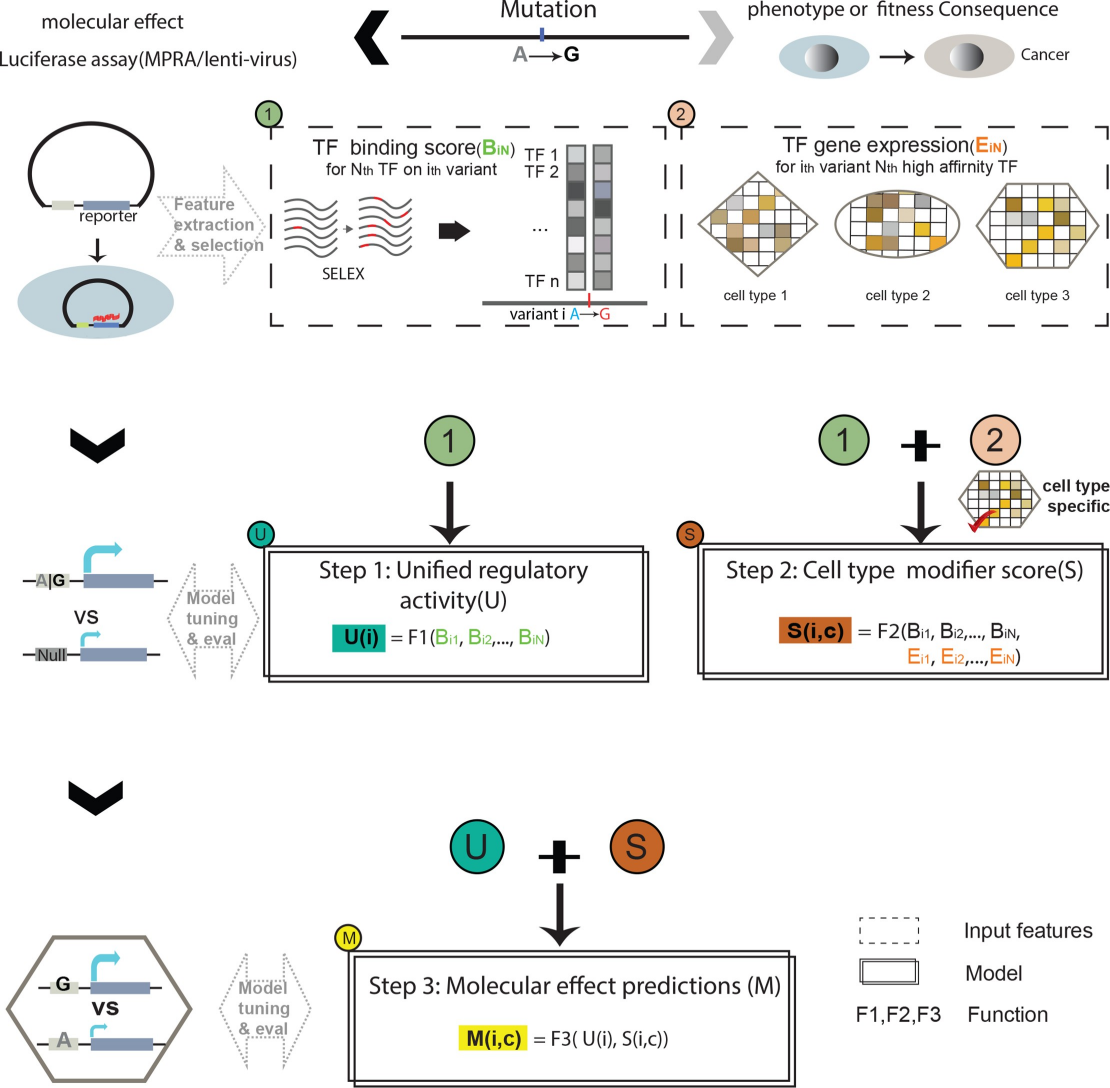
Overall flow of GRAM. The model predicts functional effects given the genotype in three steps: the first step predicts a universal regulatory activity using TF binding features; the second step predicts a cell type-specific modifier score using the TF binding score and expression profiles; the final step integrates the results from the previous two steps to predict the expression-modulating effect of the variant.

As described in Fig 1, our GRAM model is implemented in three steps: (i) prediction of the universal regulatory consequences of an element with variant using the SELEX TF binding score; (ii) prediction of a cell type modifier score in a specific cellular context by combining TF binding score with cell type-specific TF expression profiles; and (iii) estimation of the expression modulating effect in a cell type-specific context by integrating outputs from the previous two steps.

### Exploring conservation and TF binding features

We first investigated the potential of evolutionary conservation and transcription binding features as predictors. Evolutionary conservation is associated with deleterious fitness consequence and is widely used in prioritization algorithms of non-coding variants, such as PhyloP [33] and PhastCons [34] scores in LINSIGHT [28] and CADD [26], and GERP [33] score in FunSeq2 [6]. We performed comparative analyses for these three conservation features across different datasets (S1 Fig). We found that the PhastCons and PhyloP patterns of emVARs and non-emVARs are different from Human Gene Mutation Database (HGMD) [35] variants but similar to non-HGMD variants, which are thought to be benign. GERP scores show a similar pattern but have smaller variance in emVARs and non-emVARs compared to other datasets, with slightly larger values for emVARs. As we did not find differential patterns when comparing emVARs and non-emVARs, we further discovered that the correlation between logSkew and all three conservation scores was low (close to 0) by linear regression. These results suggest that the conservation scores might contribute little to the molecular effects under study that focuses on expression modulation of variants in more conserved regions with homogeneous evolutionary patterns.

**Fig 2:**
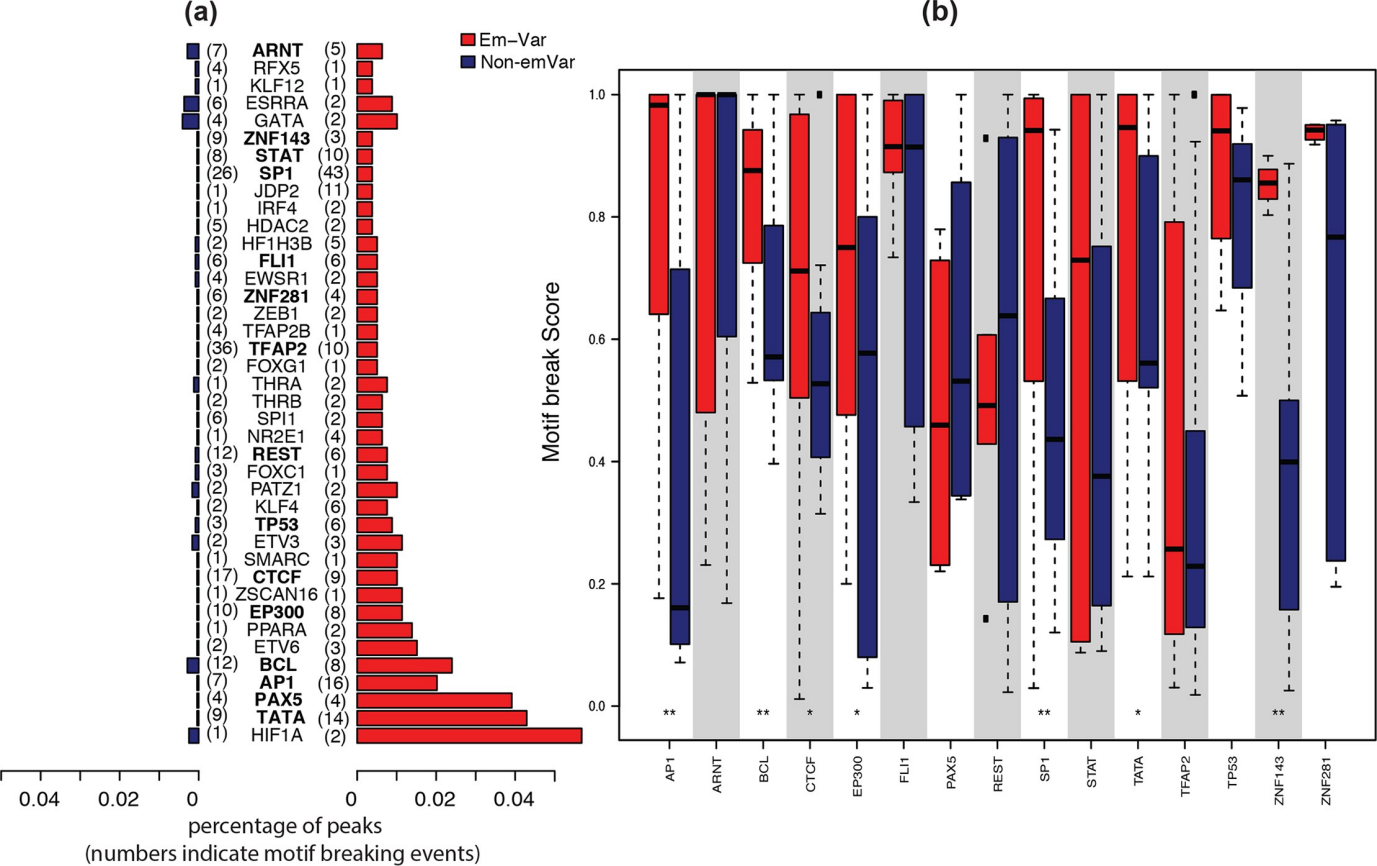
Preliminary selection of predictive features. (a) Enrichment of TF binding peaks in emVAR and non-emVAR sets. The x-axis represents a ratio of variants overlapping with the TF peaks over all variants in the same set. The TFs are sorted by p-values in hypergeometric distribution test in an decreasing order. The number in the bracket indicates the observed motif break event count. TFs with a sufficient number of observations are highlighted in bold. (b) Motif break scores in reference and alternative alleles for TFs with sufficient observed event count.

TF binding can link the molecular effect of non-coding variants to a cascade of a regulatory network, which is thought to be an important contributing factor to the variants’ regulatory effects [26, 29, 36, 37]. Tewhey et al. found that the logSkew value positively associates with TF binding scores. To thoroughly evaluate the effect of TF binding, we tested TF binding peaks overlapping with the SNVs and TF motif break events in the Tewhey dataset. We annotated and analyzed the emVAR and non-emVAR variant sets with FunSeq2 [6], and found that the emVAR set had more TF binding events compared with the non-emVAR set (Fig 2A). In addition to TF binding enrichment, we examined the motif breaking scores for these TFs. After removing TFs with insufficient observations, the differences between the distributions of motif-break scores for alternative and reference alleles in emVARs are larger than those in the non-emVAR dataset (Fig 2B). According to this analysis, the emVAR set tends to have not only more TF binding events, but also larger binding alterations compared with the non-emVAR set. Our results indicate that TF binding shows high association with the expression-modulating effects of the variants and align with recent studies on the underestimated relative importance of transcription [38, 39].

### Model-based feature selection

**Fig 3:**
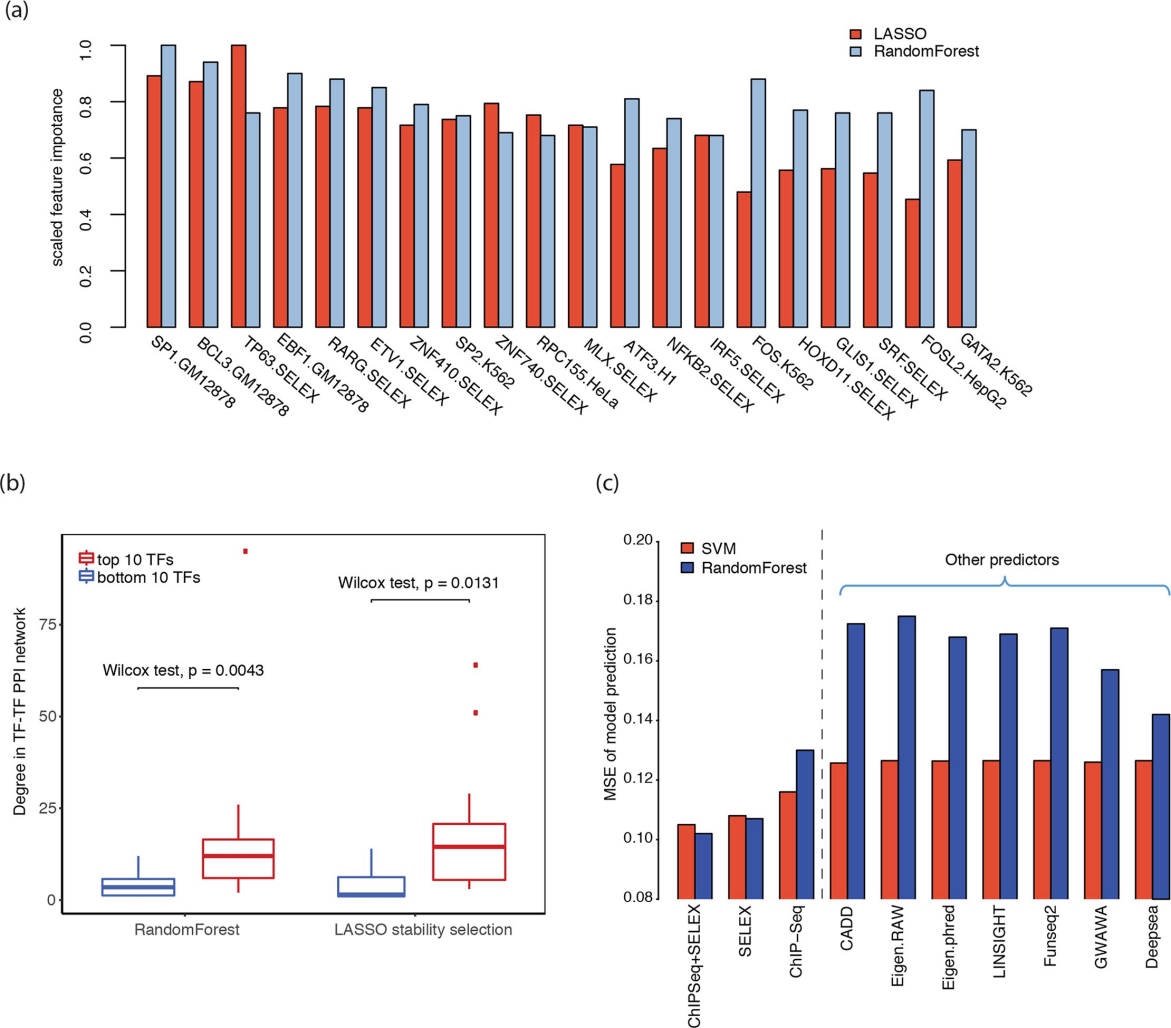
Model based feature selection. (a) Importance of the top-ranked features for SELEX- and ChIP-Seq-derived models. The features are sorted according to the mean of LASSO stability selection and Random Forest importance scores. (b) Regulatory network degree of relevant TFs for the top-ranked and bottom-ranked TFs in LASSO stability selection and Random Forest. (c) Comparison of the performance of different feature sets, including cell-line specific ChIP-Seq TF binding scores and SELEX TF binding scores, as well as features defined from previous disease-association prediction tools.

We generated a candidate training feature set from the outputs of 515 DeepBind models for TF binding, inferred from both ChIP-Seq [40] and *in vitro* SELEX assays [41], on the adjacent sequences of the variant of interest. With a comprehensive feature selection framework for selection of impactful TF binding features, we prioritized these features across models with LASSO stability selection [42] and Random Forest (shown in Fig 3A). The 20 most important features (out of 515) with respect to the mean importance across all methods is shown in decreasing order in Fig 3A. Both ChIP-Seq and SELEX DeepBind features showed high importance, with the top two being GM12878 ChIP-Seq features (SP1 and BCL3), which are cell line specific, followed by SELEX features starting with ETP63. The top-ranked impactful TFs tend to have more protein-protein interactions than the bottom-ranked TFs, indicating that the importance of a TF reflects its role in the TF-TF cascade regulatory network (Fig 3B).

Interestingly, many SELEX features, though not cell type dependent, achieved similar predictive power as cell type-specific ChIP-Seq features. We compared the predictive performances of cell type-dependent ChIP-Seq features, cell type-independent SELEX features, and a combination of both feature sets using a LASSO regressor, support vector machine (SVM) regressor and Random Forest. Incorporating ChIP-Seq-derived features, though introducing more cell type-specificity, did not boost the accuracy significantly for any of the three models (Fig 3C and S1 Table). As the availability of ChIP-Seq data is restricted to a few cell lines (S2 Fig), we instead used SELEX features to build a more generalized model that can be easily applied to different cell types.

We then used the features generated from disease-association prediction tools (CADD [43], FunSeq2 [32], DeepSEA [44], GWAVA [45], LINSIGHT [46], and Eigen [47]) to predict the same molecular effect target. As shown in Fig 3C, this analysis indicated that the prediction of disease-associated variants is not equivalent to that of expression-modulating variants.

### Building a generalized model by multi-step learning

**Fig 4:**
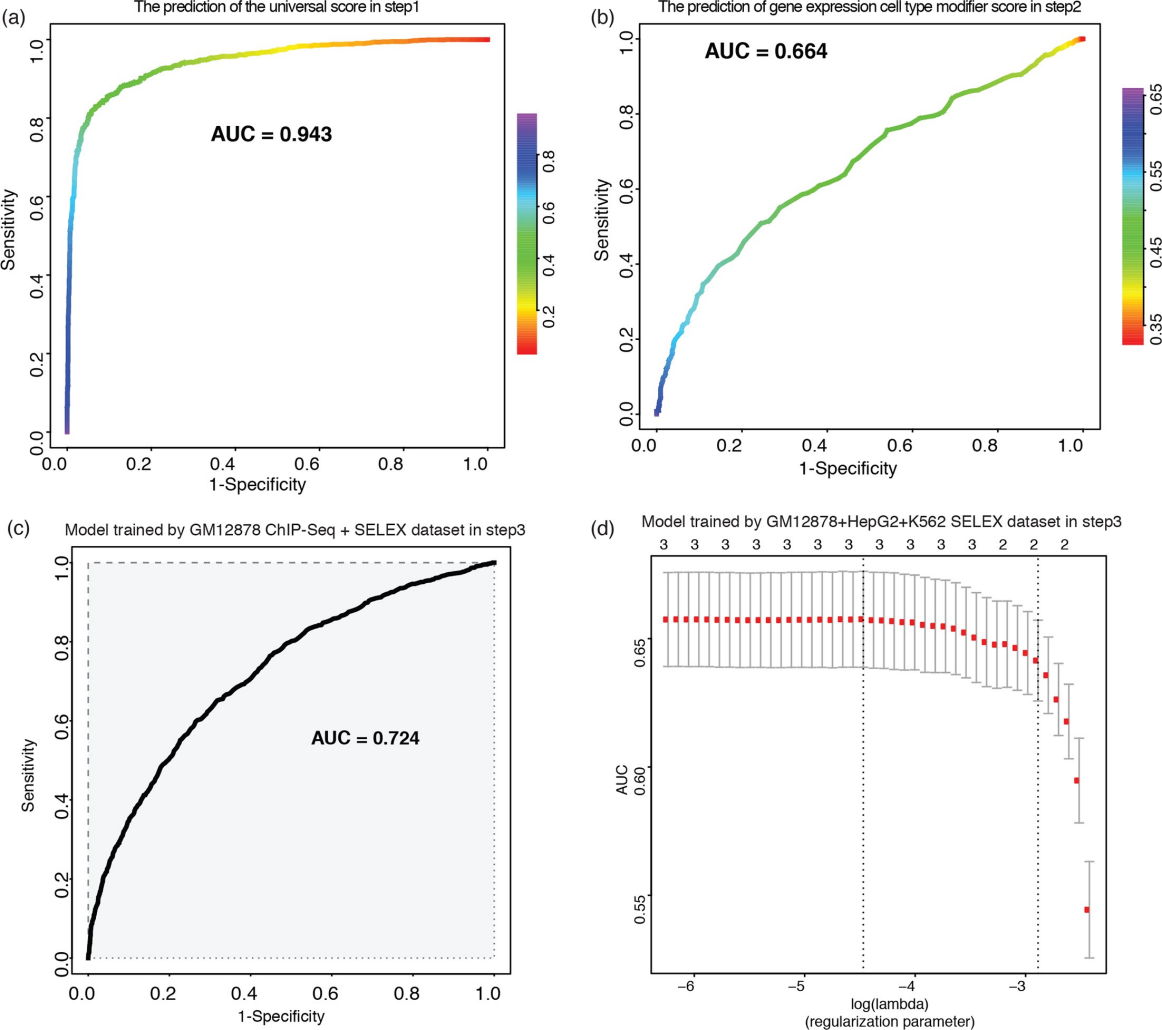
Performance of the GRAM multi-step model. (a) ROC curve for regulatory activity prediction. (b) The prediction of the cell type modifier score using TF expression profiles. (c) ROC for the model trained with both ChIP-Seq and SELEX DeepBind features on GM12878. (d) LASSO cross validation results with different regularization parameters of the final GRAM generalized model using SELEX features on a multiple cell line dataset.

Using the TF binding features from DeepBind models and the MPRA dataset from Tewhey et al. [22], we implemented our multi-step model. In the first step, we predicted the universal regulatory activity of an element with or without a variant. The 10-fold cross validation demonstrated exemplary performance of the model with an area under the receiver operating characteristic curve (AUROC) of 0.938 and an area under the precision-recall curve (AUPRC) of 0.928 (Fig 4A and S3 Fig).

In the second step, we calculated a cell-type modifier score as an indicator of the experimental assay’s cell-specific nature. Briefly, we defined the prediction target using a top and bottom quantile of Vodds (S5 Fig). Vodds is the standard deviation of log odds for each variant’s read count in MPRA, which reflects the confidence interval of log odds ratio of an experiment. Vodds shows cell line-specific patterns, as the patterns of the two B-Lymphocyte cell lines (NA12878 and NA19239) are similar while distinct from HepG2 (S4 Fig) (see Methods for details). This indicates that Vodds can capture the cell type-specific information. We also found that variants with higher Vodds tend to include more non-emVARs (Chi-square test p-value: 0.0002). Hence, the cell type modifier score defined from Vodds can be used to adjust the universal regulatory effect to a cell type-specific context.

Gene expression profiles, especially TF expression profiles, are more generally available and can represent the cellular environment. We incorporated TF gene expression and TF binding scores as features to predict the cell type modifier target, and got an AUROC of 0.66 and 0.8 (Fig 4D), respectively, using Random Forest with a 10-fold cross-validation (Fig 4B and S6 Fig).

The final step is to predict the molecular effect of a variant, i.e. whether it can significantly modulate reporter gene expression. To do this, we fed the output from the first and second step into a LASSO model, with the emVAR and non-emVAR labels as targets. We found that the AUROC of a 10-fold cross-validation for the optimal model was 0.724 (Fig 4C) and the AUPRC was 0.602, both of which are higher than the state-of-the-art method (KSM) using the same dataset (AUROC: 0.684, AUPRC: 0.478) [48].

To achieve better generalizability, we built the model with SELEX features only. We performed step (i) and (ii) on the same GM12878 dataset and another multiple-cell-line dataset (MCL dataset: GM12878 plus HepG2 plus K562). The model with cell-independent features from the SELEX assay achieved comparable performance with an AUROC = 0.664 (GM12878 only) and 0.658 (MCL dataset, Fig 4D). We use the model based on the multiple-cell-line dataset in our final GRAM model for a better generalization potential.

### Validating the GRAM model using experimental assays

**Fig 5:**
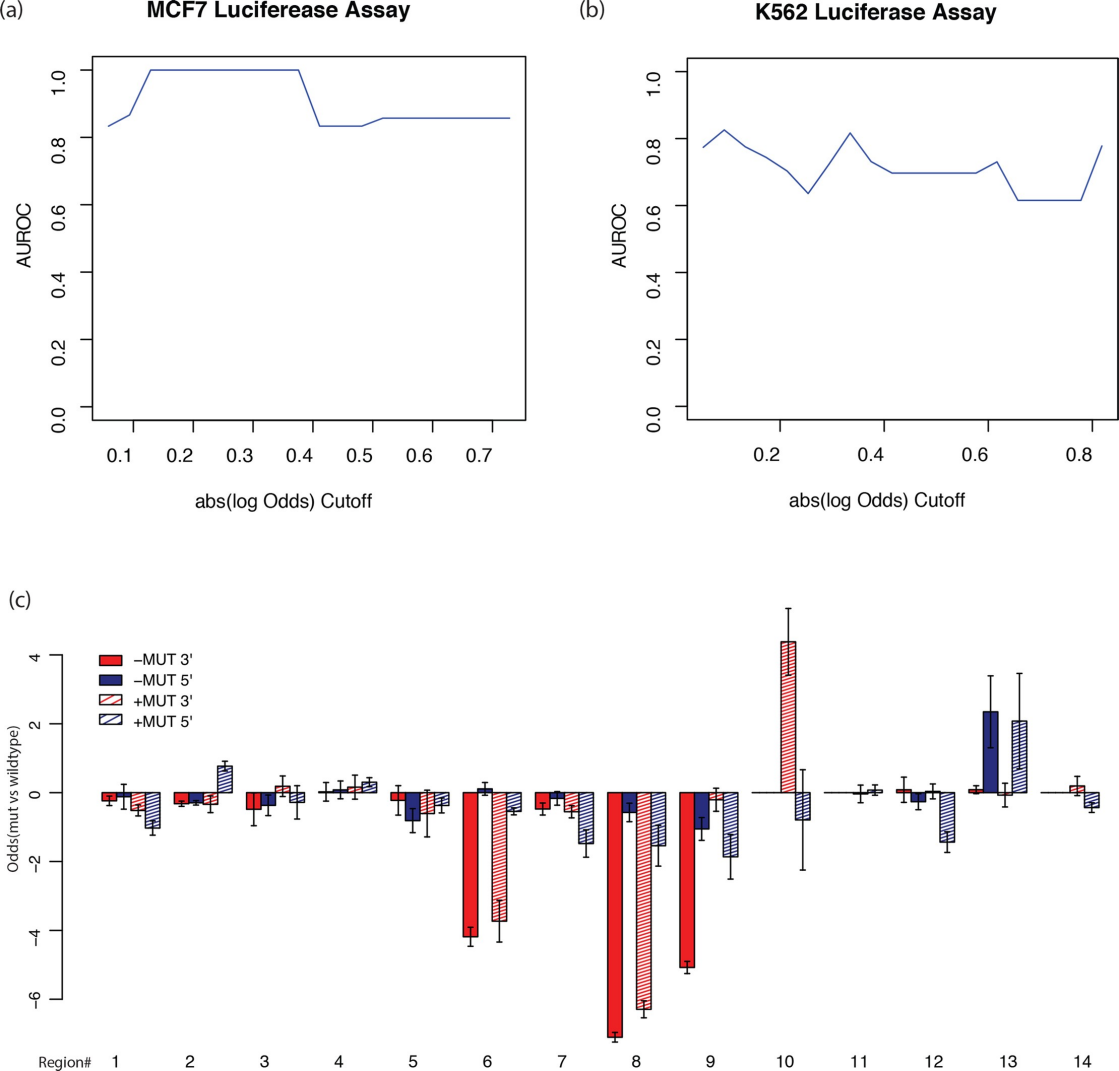
Experimental validation. (a) The AUROC value versus the different absolute log2 odds cutoff [0.5, 2.0] in the MCF7 cell line luciferase assay; The x-axis represents the log odds ratio from the luciferase assay. (b) The AUROC value versus the different absolute log2 odds cutoff [0.5, 2.0] in K562 cell line luciferase assay; (c) Experimental results (in odds ratio) for luciferase assay in K562 cell line. The 5’ terminal and 3’ terminal insertions are compared.

We next evaluated performance of the model on different cell types and assay platforms. Rather than measuring read counts as in MPRA, some other assays, such as luciferase and GFP reporter assays, measure luminescence and fluorescence readouts instead. [49, 50]. To evaluate how our model, trained with multiple cell line MPRA data, can be transferred to these assay platforms we tested its performance on luciferase assay results of eight potential regulatory elements with mutations from the MCF7 cell line [51]. To predict expression-modulating effects, we defined the significant changes between alternative and reference alleles by using an absolute log2(odds ratio) cutoff. The average AUROC value was greater than 0.8 for MCF7 (Fig 5A) and 0.67 for K562 given the an absolute log2 cutoff from 0.5 to 0.8 (Fig 5B). This indicates that our model performs very well on the luciferase assay and MPRA dataset from different cell lines, even though these assays use different measurements.

In MPRA, the element is inserted upstream (5’-terminal) of the reporter gene, but for some assays, such as STARR-Seq, the element is inserted downstream (3’-terminal). Therefore, we further tested the effect of insertion location of an element in luciferase reporters in K562 cells using 14 randomly selected elements with potential regulatory activity. As shown in Fig 5C, the 5’ terminal log odds were similar to the 3’ terminal odds for region 3, 4, 5, and 13, but showed significant differences for region 6, 8, 9, 10, and 14. The prediction of GRAM for the 5’ terminal was much better than that for the 3’-terminal insertions; the AUROC was 0.25 higher for universal regulatory activity and 0.32 higher for the expression-modulating effect prediction, indicating different mechanisms for the two ends. Therefore, GRAM model is optimal for 5’ terminal assays.

### Expression modulating effect prediction for eQTL fine mapping

As GRAM needs only gene expression and SELEX DeepBind score to predict sample-wise variants effect, it could be a flexible tool for a variety of analysis tasks. We investigated whether we could apply our GRAM model to fine-mapping of causal variants. As was described in the Methods part, we made a user-friendly pipeline GRAMMAR that could conduct the entire analysis (S9 Fig). Here we mainly focused on the task of identifying the variants that are most likely to directly modulate gene expression. For our analysis, we selected five LD blocks with known risk association with prostate cancer and high enrichment of annotated eQTL SNPs reported by Dadaev et al. [10], resulting in a set of 561 eQTL SNPs from the five LD blocks. We extracted the genotypes and gene expression data from 102 The Cancer Genome Atlas (TCGA) PRAD patients and ran GRAMMAR to get the prediction score for each allele in each patient (S4 Table).

**Fig 6:**
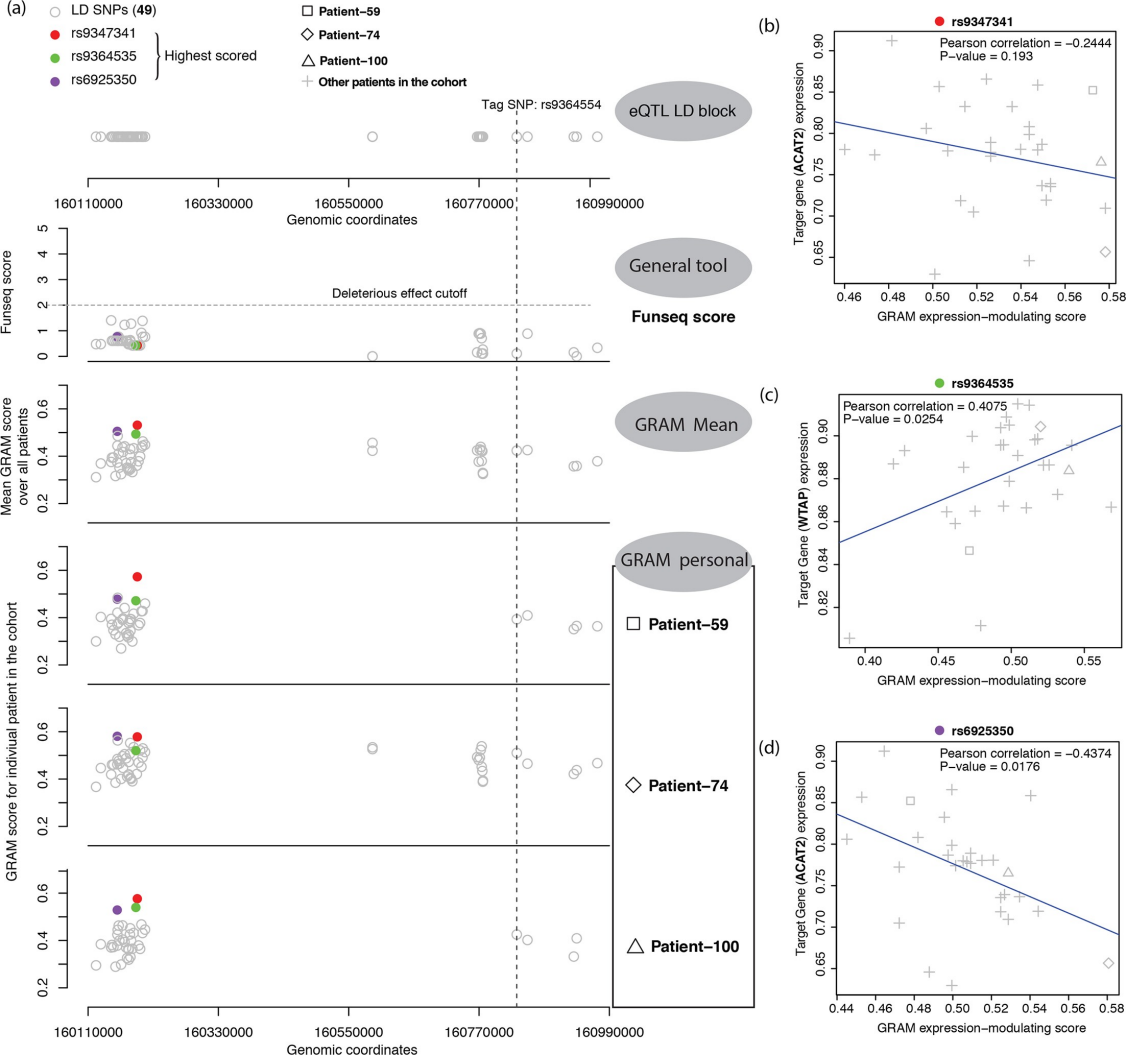
Fine mapping of variants in prostate cancer. (a) General pipeline of the fine-mapping analysis. The first panel shows the position of the variants in the LD block (chr6:160081543–161382029, tag SNP rs9364554). The second panel shows the FunSeq scores of these variants, where little variation and significance is observed. The third panel shows the average GRAM score over the patients, with three highest-average-scored variants labelled in specific colors. Personalized GRAM scores for the three highest-scored variants in three selected patients are presented subsequently. (b) ~ (d) Correlation between the GRAM score of variants with high scores and the expression of relative target genes.

In general, variants with high posterior probability (≥0.5, 130 variants), as a causal variant, reported by Dadaev et al. [10], generally have higher average GRAM scores as compared to those with lowest posterior probability (<0.5, 4260 variants) (p-value = 0.0545, S7 Fig). Specifically, we took a closer look at region chr6:160081543–161382029, tagged by GWAS SNP rs9364554 and enriched with 52 eQTL SNPs for genes including ACAT2, LOC729603, MRPL18, SLC22A3 and WTAP. All the FunSeq2 scores (maximum 1.40) are below 2, an empirical threshold for confident candidate causal SNVs. GRAMMAR, however, can pinpoint three SNV candidates with the highest average GRAM scores in this region (Fig 6A). Their GRAM scores differ in different patient samples, indicating different expression modulating effects of these SNVs under different personalized cellular contexts. Moreover, all three of the highest-scored variants show strong correlations between the GRAM expression modulating score and the expression of the related target gene and two of which are significant (p-value < 0.05) (Fig 6B–6D).

## Discussion

There has been an increasing number of computational methods that can prioritize non-coding variants. In addition, accumulating high-throughput whole-genome sequencing data have become the primary source for identifying disease-associated variants. However, we still lack an efficient prediction model for estimation of the expression-modulating effect of variants that can be universally applied to many cell lines or samples. Previous studies tend to construct one distinct model for each cell type, or predict the cell-type specificity of a variant from often very limited experimental results (e.g. ChIP-Seq) in different cell types [25, 30, 52, 53], which makes the generalization to other cell types challenging. In this study, we sought to represent the impact of cellular environments on variant function from a different perspective. We developed a multi-step generalized model called GRAM that can specifically predict the cell type-specific expression-modulating effect of a non-coding variant in the context of a particular experimental assay. Our model receives both cell type-dependent and independent input data and combines them with the same set of feature weights across different contexts, Thus, our model can be applied to any cellular context as long as cell type- or sample-specific expression data are provided.

In this study, we aim to precisely define the expression-modulating effect as a function of the predictive variables extracted from genomic data. In line with results from recent studies [38, 39], a wide array of transcription-related features demonstrated high predictive power. In contrast, three selected evolutionary features demonstrated low predictive power on used datasets. This pattern is likely due to the limited variety in evolutionary patterns in the training data and also stems from the nature of GRAM, which focuses on predicting expression-modulation effects. These effects are part of the many that are related to sequence conservation [54, 55]. In other words, the purpose of our model is to enable precise downstream analysis of molecular effects of variants in a highly conserved region, where we would not expect conservation scores to provide more additional information. We further selected a variety of TF binding features that could be useful for predicting variant effects and used direct measurements from TF binding scores and implemented a straightforward LASSO regression to assess the importance of each feature. We found that in vitro SELEX TF features (aka non-cell-specific features) achieve the highest predictive performance, a result further validated by SVM and Random Forest models trained in parallel.

We cannot ignore the cell type-specific context when predicting a variant’s effects. Usually, a model can achieve cell type-specificity in two different ways: 1) building an independent model for each cell line, or 2) building one unified model that can accept and handle specific input data from any cell lines or samples. Which strategy to use depends on the availability of the dataset and the demand for model transferability. Our model uses the second strategy, in which cell type-specific information is incorporated as an input feature and the model learns the same set of feature weights across multiple cell lines. For such a unified model, features like histone modification and TF ChIP-Seq would limit its transferability because these features may not be available for many other cell types or samples. Thus, we would prefer features that are more easily available, such as gene expression profiles. Here, we built the model using cell type-dependent gene expression and cell type-independent TF *in vitro* SELEX features; thus, the model can be more easily applied to various different samples and cell lines. SELEX features represent general binding strength of the TFs on the region of interest, and gene expression profiles can represent the specific cellular context.

The three-step GRAM model predicts the expression-modulating effects of variants by integrating two intermediate predictive targets: universal regulatory activity and cell type modifier score. The universal regulatory activity reflects the general regulatory effect of an element with or without a mutation in a vector-based assay without considering cell type-specific chromatin contexts or epigenomics information. Next, we modeled the cellular environment related to gene regulation with a cell type modifier score, derived from cell type-specific TF expression levels, to adjust the universal regulatory effect in the final step of the prediction model, greatly improving the performance.

GRAM performed well in validations on MPRA and luciferase assay, even across different cell types. In addition to target validations, our tool enables detailed exploration of the sensitivity of these methods and the impact of vector construct. The insertion position of the element affected the outcome of the assay, which may correspond to different types of regulatory elements. Because our model is trained on 5’-terminal insertion data, the prediction is consistent with outcomes from the same position, but not for 3’-terminal assay results. This indicates different mechanisms for two insertion positions: the assay with an element inserted upstream of a reporter gene may detect either the promoter or enhancer activity of the element. However, if the element is inserted downstream of the gene’s transcriptional start site or the 3’ terminal in the assay, the reporter readout may be specifically to the enhancer activity of the element. Large-scale experimental validation is required to further elucidate the underlying mechanisms.

Our GRAM model can be further applied to fine mapping of functional SNVs. Particularly, the prediction results of GRAM could aid in the identification of variants that are most likely to directly modulate gene expression in a fine-mapping study. In addition, the impact of variants on gene regulation could vary across different cell types or individuals depending on differential transcriptional factor activity, which is represented by the expression level of TFs in our model. Based on this consideration, our model could potentially be used to evaluate the molecular effect of variants in a sample-specific manner. Given a group of patients with paired genotype and gene expression data, we could evaluate for each patient the expression-modulating effect of the variants of interest, which can be used to: 1) evaluate the patient-specific expression modulating effect for each variant; 2) identify distinct expression modulating patterns among the patient population; and 3) evaluate the overall variant effects by integrating results from different patients. Such knowledge could potentially contribute to our understanding of the molecular mechanism underlying disease-association of variants, and guide the characterization of patient-specific candidate variants for personalized diagnosis, prognosis and medical treatments.

In summary, our GRAM model will be a useful tool for elucidating the underlying patterns of variants that modulate expression in a cell type- and tissue-specific context, and can be further applied to different samples of the same cell type or tissue. By leveraging the accumulating data generated from multiple cell lines, we can further improve for in-depth investigation in the future. We will keep abreast with the growing availability of comprehensive datasets and further expand our analyses.

## Methods

### Dataset

We downloaded the dataset from R. Tewhey et al.’s paper [22, 23]. From about 79K tested elements, we only kept variants for which either reference or alternative allele elements show regulatory activity. This reduced the set to 3,222 SNVs in the GM12878 cell line and 1124 SNVs in the HepG2 cell line. Each SNV was extended in both directions by 74bp, for a total of 149bp. We used another dataset from Ulirsch 2016 [17], which included 2,756 variants tested in the K562 cell line.

The protein-protein interaction network used in our downstream analysis was constructed by merging all interaction pairs identified by BioGrid [56], STRING [57] and InBio Map [58].

### Feature extraction

GERP features were extracted using the FunSeq2 annotation pipeline, which averages over the whole genome-scale GERP score over the elements. We downloaded phyloP [33] and Phastcons [34] scores from the UCSC genome browser data portal (http://hgdownload-test.cse.ucsc.edu/goldenPath/hg19/).

We performed motif enrichment analysis using a hypergeometric test. To compare the motif break and gain scores, we removed the TFs that covered less than two variants for either emVARs or non-emVARs from the list of 40 TFs with the highest p-values in hypergeometric test. Then, we performed a Wilcoxon test on the motif break score.

Motif break and motif gain scores were calculated using FunSeq2. We also calculated the motif score using DeepBind [37] with both the SELEX and ChIP-Seq motif models. SELEX motif models were identified from *in vitro* systematic evolution of ligands by an exponential enrichment (SELEX) binding assay. ChIP-Seq models were inferred from sequences of TF binding sites from different cell lines. A total of 515 motif models were investigated (S2 Table).

### Model-based feature selection

To examine the importance of features, we compared different metrics learned from various models including LASSO stability selection [42] and Random Forest regression. The feature importance for each selection method was scaled to [0, 1]; we took the mean of all the selection methods to represent the overall ranking.

We compared our models’ mean standard error (MSE) with CADD, Eigen, LINSIGHT, FunSeq2, GWAVA, and DeepSea. Features from the above tools were collected and tested using both SVR and Random Forest regression with three different input feature sets: SELEX-based features, ChIP-Seq-based features, and SELEX- and ChIP-Seq-based features combined. For other variant prioritization tools, we use their outputs as features to train the SVR and Random Forest models to predict the logSkew value.

### GRAM–multi-step generalized model

We labeled emVARs as positive and non-emVARs as negative classes following the definition of [22], where ‘expression modulating’ means having a molecular effect that significantly increases or decreases regulatory activities. We calculated the emVAR and non-emVAR for both HepG2, GM12878 and K562 cell lines from [17] [22]. For emVAR and non-emVAR, we further filtered using logSkew with an absolute value >0.5849 (skew > 1.5). In total, we used 3,222 data records, including 799 positives and 2,423 negatives.

We built a three-step GRAM model (Table 1). Step 1 predicts the universal element regulatory activity *U* for both reference and alternative alleles. The ground-truth of regulatory activity is determined from results of experimental assay platforms, like a luciferase assay or MPRA. In these assays, an element inserted into a plasmid, either with or without a mutation, is characterized with regulatory activity if the fold change between the vector with the inserted element and the control is larger than a statistically significant cutoff. Specifically, the predictive target is defined as follows: for the MPRA study, where expression level of the reporter gene is directly measured, a statistical test based on DESeq2 was used to indicate whether the expression change is significant; for the luciferase assay, we regarded a testing element that has a fold change of fluorescence level greater than 1.5 or 2 compared to control (like eGFP) as a regulatory element. The predictive variable is the TF binding score from reference to alternative allele, which is estimated by DeepBind. A Random Forest classifier was then trained to predict the universal regulatory activity. The predicted log odds of probability between the reference and alternative allele was calculated as $log2\left(\frac{U(i_{mut})}{1-U(i_{mut})}/\frac{U(i_{wt})}{1-U(i_{wt})}\right)$.

**Table 1:** Pseudocode of GRAM.

i: variant id
j: TF id
V: the total number of variants
N: the total number of TF
*B*_*ij*_: TF j binding score on variant *i*
*E*_*ij*_: Expression of *j*-th high-affinity TF on variant *i*
c: cell type or sample
*U*_*i*_: Universal score for each i for for variant *i*
*S*_*b*_(*i*,*c*): TF binding cell modifier score for variant *i* in sample c
*S*_*e*_(*i*,*c*): gene expression cell modifier score for variant *i* in sample c
*M*(*i*,*c*): molecular effect score for variant *i* in sample c
*λ*: the penalty term for the L1-regularization
*b*: the vector of coefficients, including *b*_*u*_,*b*_*s*1_,*b*_*s*2_,*b*′ for universal score, tf binding and gene expression modifier score and noise repectively.
Step1: simple Universal score to be a regulatory element using randomForest classifier, *U*(*i*)∈[0,1]
*U*(*i*) = *F*1(*B*_*i*1_,*B*_*i*2_,…,*B*_*iN*_),
Step2: TF binding and gene expression cell type modifier score, *S*(*i*,*c*)∈[0,1] using randomForest
*S*_*b*_(*i*,*c*) = *F*2(*B*_*i*1_,*B*_*i*2_,…,*B*_*iN*_)
*S*_*e*_(*i*,*c*) = *F*2(*E*_*i*1_,*E*_*i*2_,…,*E*_*iN*_)
Step3: molecular effect score, *S*(*i*,*c*)∈[0,1]
$\hat{U}(i_{mut})$ and $\hat{U}(i_{wt})$ are predicted from Step 1
$\hat{S_{b}}(i,c)$ and $\hat{S_{e}}(i,c)$ are predcited from Step2
$\hat{Odds}(i)=abs(log2(\frac{\hat{U}(i_{mut})}{1-\hat{U}(i_{mut})}/\frac{\hat{U}(i_{wt})}{1-\hat{U}(i_{wt})})),\hat{Odds}(i)\in [0,\propto ]$
$M(i,c)=F3(\hat{odds}(i)$, $\hat{S_{b}}(i,c),\hat{S_{e}}(i,c))=\frac{1}{1+e^{-1*(b_{u}\hat{Odds}(i)+b_{s1}\hat{S_{b}}(i,c)+b_{s2}\hat{S_{e}}(i,c)+b\prime }}$, link function: logit
Objective function: ${min}_{b}\frac{1}{2V}{\left\|\hat{F}-F\right\|}_{2}^{2}+\lambda {\left\|b\right\|}_{1}$, b = <*b*_*u*_,*b*_*s*1_,*b*_*s*2_,*b*′>

Time complexity for training: The complexity analysis for both Random Forest and LASSO depend on the implementation. Simply, the Random Forest worse case training cost is O(MK*N*^2^*logN*) [59], where *N* is the total number of rows, K is the number of split features for Random Forest, and M is the number of trees; the time complexity of LASSO is O(*K*^2^*N*) and almost linearly in N when *K*≪*N*, where *N* is the total number of rows, K is the number of features [60].

Step 2 predicts the gene expression and TF binding cell type modifier scores. The cell type modifier score is defined according to the cell specificity of the experimental assay. For each variant, an MPRA experiment is performed on both the reference and alternative alleles, each paired with a null-control, resulting in a 2x2 categorical table of read counts in the MPRA experiments. The standard deviation of log(odds) of the categorical table (n1, n2, n3,n4 for the average reads count, Table 2) is calculated as $\sqrt{\frac{1}{n1}+\frac{1}{n2}+\frac{1}{n3}+\frac{1}{n4}}$. For three different cell lines, GM12878, GM19239, and HepG2, we constructed a vector of Vodds values for all the variants that are tested. By comparing principal component loading of the Vodds from three cell lines, we found that the two GM cell lines are closer to each other relative to HepG2 (S3 Fig), which indicates that Vodds could reflect cell type information. We then further compared two groups of variants above the top quartile and below the bottom quartile of Vodds in GM12878, and found that there were more non-emVAR variants in the top quantile group, which indicates that Vodds are also associated with the molecular effects of the variants. Based on these observations, we used the top and bottom quartile variants as positive and negative training sets, respectively, to predict the cell type modifier target.

**Table 2:** The 2x2 categorical matrix for computation of Vodds.

Reads	Reference	Alternative
Assay	n1	n3
Null-control	n2	n4

The TF expression profiles were used as input features for the prediction of the cell type modifier class. For each mutation, we re-ordered the expression of TFs based on their binding scores. Given 258 TFs with a DeepBind SELEX model score S for 3,222 SNVs, the TF expression matrix for each variant was adjusted and re-ordered using the rank of SELEX binding scores of the TFs bound to these SNVs’ region. For each variant, this results in a vector reflecting the expression of TFs relative to their binding strengths. That is, the first value in the vector represents the adjusted expression of the most influential TF bound to this region, i.e. the one with highest rank of binding scores, and so forth, regardless of what the TFs actually are. We then used the TF binding score and re-ordered gene expression to predict the cell type modifier label.

The final model predicts the molecular effect of a variant using the estimated universal odds ratio and cell type modifiers from the two previous Steps. A LASSO model was used for the prediction. The LASSO model trained with L1 regularization is more robust and tolerant to noise. To achieve optimal predictive performance, we chose the regularization parameter lambda λ that gives minimal mean cross-validated error.

### Cross-validation

We hold out one-fold of same variants for all steps and perform a 10-fold cross-validation (S8 Fig). We first randomly permutate all the data by rows (variants), and split them into ten evenly distributed subsets *T* (1, 2…, 10). We then iteratively hold out a subset *T*_*i*_ (*i* = 1, 2…, or 10), and make sure *T*_*i*_ are not used for training in any steps. We trained the model using the remaining subset *T*_−*i*_ (−*i*: excluding *i*), and predicted the results of *T*_*i*_ to get $\hat{T_{i}}$. Finally, we concatenated all $\hat{T_{i}}’s$ and evaluated the performance using AUROC and AUPRC.

### Software

We integrated data processing pipelines and the final model into a software pipeline called GRAMMAR (S9 Fig), published on (https://github.com/gersteinlab/GRAM). The user provides the variant list and gene expression data of each sample. The sequences with and without the variants are then extracted from the hg19 genome and provided as input for DeepBind. The GRAM model receives the DeepBind results and gene expression data and assigns a score for each provided variant in each sample. Finally, the program outputs the sample-specific GRAM scores for each sample, along with heatmap for all variants and samples. If variants from multiple regions are provided, each region is plotted individually. The software is also made available as a fine-mapping module to the more generalized FunSeq tool (FunSeq.gersteinlab.org), taking in the variants prioritized by the first tool and outputting the subset of them that have a direct expression modulating function.

### Fine mapping

The work by Dadaev et al. [10] reported 75 different LD blocks characterized by a known GWAS risk association for prostate cancer. Some of the SNPs in these regions were found to be significantly co-localized with identified eQTLs, annotated as eQTL SNPs. For our analysis, we selected five regions with the largest number of eQTL SNPs, which in total contains 561 eQTL SNPs. Genotype and gene expression data for 102 TCGA PRAD patients were obtained from the TCGA data portal. These data were then provided to the GRAMMAR pipeline described above.

We plotted the estimated sample-wise GRAM scores for each region, and selected variants with the highest average GRAM scores as assumed causal variants for expression modulation. As a comparison, FunSeq [6] scores for each variant were also extracted based on position and allele. To analyze the impact of these variants on gene expression, we calculated the Pearson correlation between the sample-specific GRAM scores and expression of the target genes of each eQTL variant.

### Experimental validation on MCF7 cells

Each regulatory region (both reference and alternative alleles) was separately synthesized. Enhancer regions were designed to include 250bp upstream and 250bp downstream for each enhancer region based on the candidate SNV site. These regions were then cloned into the pGL4.23[luc2/minP] vector (Promega, Cat# E841A). Each candidate region was placed upstream of the minP promoter to determine the effect of each putative enhancer region on luciferase expression. In total, 100ng of each candidate construct and 100ng of Nano-luc control was co- transfected into MCF-7 cells (5,000 cells per well in DMEM media containing 10% FBS and 1% Penicillin-Streptomycin antibiotic) using the Lipofectamine 3000 reagent (Thermo Fisher, Cat# L3000001) according to the manufacturer’s instructions. Cells were incubated for 48 hrs before reading the luciferase signal using the Promega Nano-Glo luciferase kit (Promega, Cat# N1521) according to the manufacturer’s instructions.

### Model validation using MPRA data from K562 cells

#### Enhancer selection

Based on the enhancer prediction and histone mark signaling overlap, we randomly selected 14 putative regulatory elements, and then randomly picked one or two mutations based on FunSeq2 whole genome scores (http://funseq3.gersteinlab.org). Next, we used a web tool to design site-directed mutagenesis primers to introduce the target SNVs into the 14 elements. Two SNVs were introduced into each element, with only one predicted to result in a significant change in enhancer activity.

#### Reporter generation

Elements were amplified via PCR from human genomic DNA (Promega) with Platinum SuperFi polymerase (Invitrogen) and primers containing attB1 and attB2 sequences (see S3 Table). Elements were then cloned into pDONR223 using Gateway BP clonase and transformed into *E*. *coli* cells. Four colonies for each element were picked and sequenced via Sanger sequencing using the RV3 primer. One clone for each element with the correct sequence was then cloned into pDEST-hSCP1-luc or pGL4-Gateway-SCP1 using Gateway LR clonase, and luciferase reporters containing the elements were then transfected into K562 cells. pGL4-Gateway-SCP1 was a gift from Alexander Stark (Addgene plasmid # 71510) [61]. To construct a positive control for the enhancer activity assays, we cloned the widely used Rous sarcoma virus promoter that has been implied to possess enhancer activities.

#### Mutagenesis

The reference allele templates for site-directed mutagenesis were sequence-verified entry clones containing putative regulatory elements. The mutagenesis primers containing the pre-designed mutations were designed with a web tool (http://primer.yulab.org/). The mutagenesis reactions were carried out following the Clone-Seq pipeline [62]. Each mutagenesis reaction contained a reference allele template and its corresponding mutagenesis primers. The products of the mutagenesis reaction were DpnI-digested and transformed into TOP10 chemically competent cells (Invitrogen). The transformants were spread on LB-spectinomycin agar plates and incubated at 37°C overnight. Single colonies yielded from the mutagenesis were picked, propagated, and sequence-verified before they were used in downstream experiments.

#### Cell Lines

K562 cells were a gift from the Melnick lab (Weill Cornell Medicine). Cells were cultured in Iscove’s Modified Dulbecco’s Medium (Gibco) supplemented with 10% FBS and 1% Pen-Strep at 37°C with 5% CO2.

#### Luciferase assay

K562 cells were transfected with 200 ng of the above reporters and 20 ng of Renilla luciferase (pRL-CMV, Promega) in triplicate in 96-well plates with Lipofectamine 3000 (Invitrogen). At 48 hours post-transfection, luciferase activity was assayed with the Dual-Glo Luciferase Assay System (Promega).

## Supporting information

S1 Table Predictive performance of different feature sets, including cell-line specific ChIP-Seq TF binding scores and SELEX TF binding scores, using Lasso, SVM and Random Forest. (DOCX)

S2 Table All DeepBind features used in the study. (DOCX)

S3 Table Primers for 14 regions cloning in K562. (DOCX)

S4 Table Sample output of the GRAMMAR pipeline. (TXT)

S1 Fig Distribution of conservation scores among different annotation categories. (PDF)

S2 Fig Availability of different data types in ENCODE. (PDF)

S3 Fig PRC curve for regulatory activity prediction. (PDF)

S4 Fig Principal component analysis using Vodds for three cell lines: GM12878, GM19239 and HepG2. (PDF)

S5 Fig Distribution of Vodds score for GM12878. The high and low variable cell specificity class are defined by the top and bottom quantile.
(PDF)

S6 Fig The prediction of cell type modifier score using TF binding profiles. (PDF)

S7 Fig Comparison of average GRAM scores with high land low posterior probability reported by Dadaev et al. (PDF)

S8 Fig The training and cross-validation scheme. (PDF)

S9 Fig The GRAMMAR pipeline. (PDF)
