# Supplementary material for: GRAM: A GeneRAlized Model to predict the molecular effect of a non-coding variant in a cell-type specific manner
Source: PLoS Genet. 2019 Aug 30;15(8):e1007860. doi: 10.1371/journal.pgen.1007860 (PMC6742416; doi:10.1371/journal.pgen.1007860)
Supplement: S1 Table — (DOCX) [file pgen.1007860.s001.docx]

**S1 Table** Predictive performance of different feature sets, including cell-line specific ChIP-Seq TF binding scores and SELEX TF binding scores, using Lasso, SVM and Random Forest.

| **MSE** | **Lasso 1se** | **SVM** | **Random Forest** |
| --- | --- | --- | --- |
| ChIPseq+SELEX | 0.106 | 0.105 | 0.102 |
| ChIPseq | 0.124 | 0.116 | 0.130 |
| SELEX | 0.111 | 0.108 | 0.107 |
