# Supplementary material for: GRAM: A GeneRAlized Model to predict the molecular effect of a non-coding variant in a cell-type specific manner
Source: PLoS Genet. 2019 Aug 30;15(8):e1007860. doi: 10.1371/journal.pgen.1007860 (PMC6742416; doi:10.1371/journal.pgen.1007860)
Supplement: S2 Table — (DOCX) [file pgen.1007860.s002.docx]

**S2 Table** All DeepBind features used in the study.

| **ID** | **TF** | **Experiment** | **Cell type** |
| --- | --- | --- | --- |
| D00290.003 | ALX3 | SELEX | DBD |
| D00292.001 | ALX4 | SELEX | DBD |
| D00293.003 | ARNTL | SELEX | DBD |
| D00295.002 | ARX | SELEX | DBD |
| D00296.006 | AR | SELEX | FL |
| D00298.003 | ATF4 | SELEX | DBD |
| D00299.003 | ATF7 | SELEX | DBD |
| D00302.003 | BARHL2 | SELEX | DBD |
| D00303.002 | BARX1 | SELEX | DBD |
| D00304.003 | BATF3 | SELEX | DBD |
| D00305.003 | BCL6B | SELEX | DBD |
| D00306.003 | BHLHA15 | SELEX | DBD |
| D00308.003 | BHLHB2 | SELEX | DBD |
| D00309.002 | BHLHB3 | SELEX | FL |
| D00310.002 | BHLHE22 | SELEX | DBD |
| D00311.003 | BHLHE23 | SELEX | DBD |
| D00312.002 | BHLHE41 | SELEX | FL |
| D00313.002 | BSX | SELEX | DBD |
| D00314.001 | CART1 | SELEX | DBD |
| D00315.003 | CDX1 | SELEX | DBD |
| D00316.003 | CDX2 | SELEX | DBD |
| D00317.003 | CEBPB | SELEX | DBD |
| D00318.001 | CEBPD | SELEX | DBD |
| D00319.003 | CEBPE | SELEX | DBD |
| D00320.001 | CEBPG | SELEX | DBD |
| D00321.003 | CENPB | SELEX | FL |
| D00322.002 | CLOCK | SELEX | DBD |
| D00323.002 | CPEB1 | SELEX | FL |
| D00324.003 | CREB3L1 | SELEX | DBD |
| D00326.002 | CREB3 | SELEX | FL |
| D00328.003 | CTCF | SELEX | FL |
| D00329.002 | CUX1 | SELEX | DBD |
| D00330.002 | CUX2 | SELEX | DBD |
| D00331.006 | DBP | SELEX | FL |
| D00332.003 | DLX1 | SELEX | DBD |
| D00335.003 | DLX2 | SELEX | DBD |
| D00336.001 | DLX3 | SELEX | DBD |
| D00337.001 | DLX4 | SELEX | DBD |
| D00338.002 | DLX5 | SELEX | FL |
| D00339.002 | DLX6 | SELEX | DBD |
| D00340.003 | DMBX1 | SELEX | DBD |
| D00341.003 | DPRX | SELEX | DBD |
| D00342.003 | DRGX | SELEX | DBD |
| D00343.003 | DUXA | SELEX | DBD |
| D00344.002 | E2F1 | SELEX | DBD |
| D00345.002 | E2F2 | SELEX | DBD |
| D00346.003 | E2F3 | SELEX | DBD |
| D00347.003 | E2F4 | SELEX | DBD |
| D00348.003 | E2F7 | SELEX | DBD |
| D00349.002 | E2F8 | SELEX | DBD |
| D00350.002 | EBF1 | SELEX | FL |
| D00351.001 | EGR1 | SELEX | DBD |
| D00351.006 | EGR1 | SELEX | FL |
| D00353.005 | EGR2 | SELEX | FL |
| D00354.002 | EGR3 | SELEX | DBD |
| D00355.002 | EGR4 | SELEX | DBD |
| D00356.005 | ELF1 | SELEX | DBD |
| D00357.002 | ELF3 | SELEX | DBD |
| D00359.004 | ELF5 | SELEX | FL |
| D00360.004 | ELK1 | SELEX | FL |
| D00361.001 | ELK3 | SELEX | DBD |
| D00363.003 | ELK4 | SELEX | DBD |
| D00364.002 | EMX1 | SELEX | DBD |
| D00365.003 | EMX2 | SELEX | DBD |
| D00366.003 | EN1 | SELEX | DBD |
| D00368.007 | EN2 | SELEX | FL |
| D00369.003 | EOMES | SELEX | DBD |
| D00370.003 | ERF | SELEX | DBD |
| D00371.003 | ERG | SELEX | DBD |
| D00372.003 | ESR1 | SELEX | DBD |
| D00374.003 | ESRRA | SELEX | DBD |
| D00375.003 | ESRRB | SELEX | DBD |
| D00376.003 | ESRRG | SELEX | FL |
| D00377.001 | ESX1 | SELEX | DBD |
| D00378.007 | ETS1 | SELEX | FL |
| D00379.001 | ETV1 | SELEX | DBD |
| D00380.002 | ETV2 | SELEX | DBD |
| D00381.003 | ETV3 | SELEX | DBD |
| D00382.003 | ETV4 | SELEX | DBD |
| D00383.002 | ETV5 | SELEX | DBD |
| D00384.002 | ETV6 | SELEX | FL |
| D00385.002 | EVX1 | SELEX | DBD |
| D00386.001 | EVX2 | SELEX | DBD |
| D00387.001 | FEV | SELEX | DBD |
| D00388.003 | FIGLA | SELEX | DBD |
| D00389.005 | FLI1 | SELEX | FL |
| D00390.002 | FOXB1 | SELEX | DBD |
| D00392.002 | FOXC1 | SELEX | DBD |
| D00393.003 | FOXC2 | SELEX | DBD |
| D00394.003 | FOXD2 | SELEX | DBD |
| D00395.002 | FOXD3 | SELEX | DBD |
| D00396.003 | FOXG1 | SELEX | DBD |
| D00397.003 | FOXI1 | SELEX | FL |
| D00398.001 | FOXJ2 | SELEX | DBD |
| D00400.003 | FOXJ3 | SELEX | DBD |
| D00401.003 | FOXK1 | SELEX | DBD |
| D00403.001 | FOXL1 | SELEX | FL |
| D00404.002 | FOXO1 | SELEX | DBD |
| D00405.003 | FOXO3 | SELEX | FL |
| D00406.002 | FOXO4 | SELEX | DBD |
| D00407.003 | FOXO6 | SELEX | DBD |
| D00408.003 | FOXP3 | SELEX | DBD |
| D00409.003 | GABPA | SELEX | FL |
| D00410.003 | GATA3 | SELEX | DBD |
| D00411.003 | GATA4 | SELEX | DBD |
| D00412.002 | GATA5 | SELEX | DBD |
| D00413.003 | GBX1 | SELEX | DBD |
| D00416.005 | GBX2 | SELEX | FL |
| D00417.005 | GCM1 | SELEX | FL |
| D00418.003 | GCM2 | SELEX | DBD |
| D00419.003 | GLI2 | SELEX | DBD |
| D00420.004 | GLIS1 | SELEX | DBD |
| D00421.003 | GLIS2 | SELEX | DBD |
| D00422.003 | GLIS3 | SELEX | DBD |
| D00423.005 | GMEB2 | SELEX | DBD |
| D00424.002 | GRHL1 | SELEX | DBD |
| D00425.002 | GSC2 | SELEX | DBD |
| D00426.003 | GSC | SELEX | FL |
| D00427.003 | GSX1 | SELEX | DBD |
| D00428.002 | GSX2 | SELEX | DBD |
| D00429.003 | HES5 | SELEX | DBD |
| D00430.003 | HES7 | SELEX | DBD |
| D00431.002 | HESX1 | SELEX | DBD |
| D00432.003 | HEY1 | SELEX | DBD |
| D00433.003 | HEY2 | SELEX | DBD |
| D00435.003 | HIC2 | SELEX | DBD |
| D00436.002 | HINFP1 | SELEX | FL |
| D00438.003 | HLF | SELEX | FL |
| D00440.003 | HMX1 | SELEX | DBD |
| D00441.003 | HMX2 | SELEX | DBD |
| D00442.003 | HMX3 | SELEX | DBD |
| D00443.001 | HNF1A | SELEX | FL |
| D00444.001 | HNF1B | SELEX | FL |
| D00446.005 | HNF4A | SELEX | FL |
| D00447.002 | HOMEZ | SELEX | DBD |
| D00448.002 | HOXA10 | SELEX | DBD |
| D00450.003 | HOXA13 | SELEX | DBD |
| D00451.002 | HOXA1 | SELEX | DBD |
| D00453.002 | HOXA2 | SELEX | DBD |
| D00454.003 | HOXB13 | SELEX | DBD |
| D00455.001 | HOXB2 | SELEX | DBD |
| D00456.001 | HOXB3 | SELEX | DBD |
| D00457.003 | HOXB5 | SELEX | DBD |
| D00459.003 | HOXC10 | SELEX | DBD |
| D00460.003 | HOXC11 | SELEX | DBD |
| D00461.003 | HOXC12 | SELEX | DBD |
| D00462.002 | HOXC13 | SELEX | DBD |
| D00463.003 | HOXD11 | SELEX | DBD |
| D00464.001 | HOXD12 | SELEX | DBD |
| D00465.003 | HOXD13 | SELEX | DBD |
| D00468.003 | HOXD8 | SELEX | DBD |
| D00470.005 | HSF1 | SELEX | FL |
| D00471.002 | HSF2 | SELEX | DBD |
| D00472.003 | HSF4 | SELEX | DBD |
| D00473.003 | HSFY2 | SELEX | DBD |
| D00474.003 | ID4 | SELEX | DBD |
| D00475.003 | IRF3 | SELEX | FL |
| D00476.002 | IRF4 | SELEX | FL |
| D00477.003 | IRF5 | SELEX | FL |
| D00478.003 | IRF7 | SELEX | DBD |
| D00479.006 | IRF8 | SELEX | FL |
| D00480.002 | IRF9 | SELEX | FL |
| D00481.003 | IRX2 | SELEX | DBD |
| D00483.001 | IRX5 | SELEX | DBD |
| D00484.003 | ISL2 | SELEX | DBD |
| D00485.004 | ISX | SELEX | FL |
| D00487.003 | JDP2 | SELEX | DBD |
| D00488.003 | KLF13 | SELEX | FL |
| D00489.002 | KLF14 | SELEX | DBD |
| D00490.003 | KLF16 | SELEX | DBD |
| D00491.003 | LBX2 | SELEX | DBD |
| D00492.003 | LEF1 | SELEX | DBD |
| D00493.003 | LHX2 | SELEX | DBD |
| D00495.003 | LHX6 | SELEX | FL |
| D00497.002 | LHX9 | SELEX | DBD |
| D00498.002 | LMX1A | SELEX | DBD |
| D00499.001 | LMX1B | SELEX | DBD |
| D00501.003 | MAFF | SELEX | DBD |
| D00502.002 | MAFG | SELEX | FL |
| D00503.004 | MAFK | SELEX | DBD |
| D00504.002 | MAX | SELEX | DBD |
| D00505.003 | MEF2A | SELEX | DBD |
| D00506.003 | MEF2B | SELEX | FL |
| D00507.002 | MEF2D | SELEX | DBD |
| D00508.004 | MEIS1 | SELEX | DBD |
| D00510.003 | MEIS2 | SELEX | DBD |
| D00512.001 | MEIS3 | SELEX | DBD |
| D00513.002 | MEOX1 | SELEX | FL |
| D00515.002 | MEOX2 | SELEX | DBD |
| D00516.002 | MESP1 | SELEX | DBD |
| D00517.002 | MGA | SELEX | DBD |
| D00518.002 | MIXL1 | SELEX | FL |
| D00519.002 | MLXIPL | SELEX | FL |
| D00520.002 | MLX | SELEX | FL |
| D00521.003 | MNT | SELEX | DBD |
| D00522.002 | MNX1 | SELEX | DBD |
| D00523.003 | MSC | SELEX | FL |
| D00524.002 | MSX1 | SELEX | DBD |
| D00525.002 | MSX2 | SELEX | DBD |
| D00527.003 | MTF1 | SELEX | DBD |
| D00528.001 | MYBL1 | SELEX | DBD |
| D00529.001 | MYBL2 | SELEX | DBD |
| D00530.003 | MYF6 | SELEX | FL |
| D00531.003 | NEUROD2 | SELEX | FL |
| D00532.003 | NEUROG2 | SELEX | DBD |
| D00533.003 | NFAT5 | SELEX | DBD |
| D00534.003 | NFATC1 | SELEX | FL |
| D00535.003 | NFE2 | SELEX | DBD |
| D00536.003 | NFIA | SELEX | FL |
| D00537.001 | NFIB | SELEX | FL |
| D00538.001 | NFIL3 | SELEX | DBD |
| D00539.001 | NFIX | SELEX | FL |
| D00540.002 | NFKB1 | SELEX | DBD |
| D00541.001 | NFKB2 | SELEX | DBD |
| D00542.005 | NHLH1 | SELEX | FL |
| D00543.005 | NKX2-3 | SELEX | FL |
| D00544.005 | NKX2-8 | SELEX | FL |
| D00546.003 | NKX3-1 | SELEX | FL |
| D00547.003 | NKX3-2 | SELEX | DBD |
| D00549.002 | NKX6-1 | SELEX | DBD |
| D00550.002 | NKX6-2 | SELEX | DBD |
| D00551.002 | NOTO | SELEX | DBD |
| D00552.002 | NR2C2 | SELEX | DBD |
| D00553.003 | NR2E1 | SELEX | FL |
| D00554.003 | NR2F1 | SELEX | DBD |
| D00555.002 | NR2F6 | SELEX | DBD |
| D00556.003 | NR3C1 | SELEX | DBD |
| D00557.002 | NR3C2 | SELEX | DBD |
| D00558.002 | NR4A2 | SELEX | FL |
| D00559.001 | NRF1 | SELEX | FL |
| D00560.003 | NRL | SELEX | DBD |
| D00561.001 | OLIG1 | SELEX | DBD |
| D00562.006 | OLIG2 | SELEX | FL |
| D00563.002 | OLIG3 | SELEX | DBD |
| D00564.004 | ONECUT1 | SELEX | FL |
| D00565.002 | ONECUT2 | SELEX | DBD |
| D00566.003 | ONECUT3 | SELEX | DBD |
| D00568.001 | OTX1 | SELEX | DBD |
| D00569.002 | OTX2 | SELEX | DBD |
| D00570.002 | PAX1 | SELEX | DBD |
| D00571.002 | PAX2 | SELEX | DBD |
| D00572.002 | PAX3 | SELEX | DBD |
| D00573.002 | PAX4 | SELEX | DBD |
| D00574.002 | PAX5 | SELEX | DBD |
| D00575.003 | PAX6 | SELEX | DBD |
| D00576.005 | PAX7 | SELEX | FL |
| D00577.002 | PAX9 | SELEX | DBD |
| D00578.002 | PDX1 | SELEX | DBD |
| D00579.002 | PHOX2A | SELEX | DBD |
| D00580.005 | PHOX2B | SELEX | FL |
| D00581.002 | PITX1 | SELEX | DBD |
| D00582.001 | PITX3 | SELEX | DBD |
| D00583.002 | PKNOX1 | SELEX | DBD |
| D00585.003 | PKNOX2 | SELEX | DBD |
| D00586.004 | POU1F1 | SELEX | DBD |
| D00587.002 | POU2F1 | SELEX | DBD |
| D00589.001 | POU2F2 | SELEX | DBD |
| D00590.001 | POU2F3 | SELEX | DBD |
| D00591.002 | POU3F1 | SELEX | DBD |
| D00592.003 | POU3F2 | SELEX | DBD |
| D00593.003 | POU3F3 | SELEX | DBD |
| D00594.002 | POU3F4 | SELEX | DBD |
| D00595.001 | POU4F1 | SELEX | DBD |
| D00596.005 | POU4F2 | SELEX | FL |
| D00597.001 | POU4F3 | SELEX | DBD |
| D00598.001 | POU5F1P1 | SELEX | DBD |
| D00599.006 | POU6F2 | SELEX | FL |
| D00600.001 | PRDM1 | SELEX | FL |
| D00601.003 | PRDM4 | SELEX | FL |
| D00602.008 | PROP1 | SELEX | FL |
| D00603.003 | PROX1 | SELEX | DBD |
| D00604.004 | PRRX1 | SELEX | FL |
| D00606.002 | PRRX2 | SELEX | FL |
| D00607.006 | RARA | SELEX | FL |
| D00610.003 | RARB | SELEX | FL |
| D00611.001 | RARG | SELEX | DBD |
| D00612.001 | RAXL1 | SELEX | DBD |
| D00613.002 | RAX | SELEX | DBD |
| D00614.001 | RFX2 | SELEX | DBD |
| D00616.002 | RFX3 | SELEX | DBD |
| D00618.003 | RFX4 | SELEX | DBD |
| D00619.003 | RFX5 | SELEX | DBD |
| D00621.003 | RHOXF1 | SELEX | DBD |
| D00622.003 | RORA | SELEX | DBD |
| D00623.003 | RUNX2 | SELEX | DBD |
| D00624.007 | RUNX3 | SELEX | FL |
| D00626.005 | RXRA | SELEX | FL |
| D00628.002 | RXRB | SELEX | DBD |
| D00629.006 | RXRG | SELEX | FL |
| D00630.003 | SCRT1 | SELEX | DBD |
| D00631.002 | SCRT2 | SELEX | DBD |
| D00632.001 | SHOX2 | SELEX | DBD |
| D00634.001 | SHOX | SELEX | DBD |
| D00635.001 | SMAD3 | SELEX | DBD |
| D00636.003 | SNAI2 | SELEX | DBD |
| D00637.003 | SOX10 | SELEX | FL |
| D00639.003 | SOX14 | SELEX | DBD |
| D00640.003 | SOX15 | SELEX | FL |
| D00642.003 | SOX18 | SELEX | FL |
| D00644.003 | SOX21 | SELEX | DBD |
| D00645.006 | SOX2 | SELEX | DBD |
| D00646.002 | SOX4 | SELEX | DBD |
| D00647.002 | SOX7 | SELEX | FL |
| D00648.004 | SOX8 | SELEX | DBD |
| D00649.002 | SOX9 | SELEX | DBD |
| D00650.005 | SP1 | SELEX | DBD |
| D00651.003 | SP3 | SELEX | DBD |
| D00652.003 | SP4 | SELEX | FL |
| D00653.003 | SP8 | SELEX | DBD |
| D00654.003 | SPDEF | SELEX | DBD |
| D00655.002 | SPI1 | SELEX | FL |
| D00656.001 | SPIB | SELEX | DBD |
| D00658.003 | SPIC | SELEX | FL |
| D00659.002 | SREBF2 | SELEX | DBD |
| D00660.005 | SRF | SELEX | FL |
| D00661.003 | SRY | SELEX | DBD |
| D00662.003 | TBR1 | SELEX | DBD |
| D00663.002 | TBX15 | SELEX | DBD |
| D00664.002 | TBX19 | SELEX | DBD |
| D00665.002 | TBX1 | SELEX | DBD |
| D00666.002 | TBX20 | SELEX | DBD |
| D00667.001 | TBX21 | SELEX | DBD |
| D00668.003 | TBX2 | SELEX | FL |
| D00669.002 | TBX4 | SELEX | DBD |
| D00670.002 | TBX5 | SELEX | DBD |
| D00672.001 | TCF3 | SELEX | DBD |
| D00673.001 | TCF4 | SELEX | DBD |
| D00674.003 | TCF7L1 | SELEX | FL |
| D00677.003 | TEAD1 | SELEX | FL |
| D00678.001 | TEAD3 | SELEX | DBD |
| D00680.001 | TEF | SELEX | DBD |
| D00681.002 | TFAP2A | SELEX | DBD |
| D00682.002 | TFAP2B | SELEX | DBD |
| D00683.002 | TFAP2C | SELEX | DBD |
| D00684.005 | TFAP4 | SELEX | FL |
| D00685.003 | TFCP2 | SELEX | FL |
| D00686.002 | TFE3 | SELEX | DBD |
| D00687.001 | TFEB | SELEX | FL |
| D00688.002 | TFEC | SELEX | DBD |
| D00689.003 | TGIF1 | SELEX | DBD |
| D00690.003 | TGIF2LX | SELEX | FL |
| D00691.001 | TGIF2 | SELEX | DBD |
| D00692.002 | THRA | SELEX | FL |
| D00693.002 | THRB | SELEX | DBD |
| D00695.002 | TP63 | SELEX | DBD |
| D00697.002 | T | SELEX | FL |
| D00698.001 | UNCX | SELEX | DBD |
| D00700.001 | USF1 | SELEX | DBD |
| D00701.001 | VAX1 | SELEX | DBD |
| D00702.001 | VAX2 | SELEX | DBD |
| D00704.001 | VDR | SELEX | FL |
| D00705.003 | VENTX | SELEX | DBD |
| D00707.004 | VSX1 | SELEX | FL |
| D00708.001 | VSX2 | SELEX | DBD |
| D00709.002 | XBP1 | SELEX | DBD |
| D00710.002 | YY1 | SELEX | FL |
| D00711.003 | YY2 | SELEX | DBD |
| D00712.002 | ZBED1 | SELEX | DBD |
| D00713.003 | ZBTB49 | SELEX | DBD |
| D00714.003 | ZBTB7A | SELEX | DBD |
| D00715.002 | ZBTB7B | SELEX | FL |
| D00716.004 | ZBTB7C | SELEX | FL |
| D00719.002 | ZIC1 | SELEX | FL |
| D00721.002 | ZIC3 | SELEX | FL |
| D00722.002 | ZIC4 | SELEX | DBD |
| D00723.003 | ZNF143 | SELEX | DBD |
| D00724.003 | ZNF232 | SELEX | FL |
| D00725.004 | ZNF238 | SELEX | FL |
| D00726.003 | ZNF282 | SELEX | DBD |
| D00727.003 | ZNF306 | SELEX | FL |
| D00728.002 | ZNF410 | SELEX | DBD |
| D00729.003 | ZNF435 | SELEX | FL |
| D00730.002 | ZNF524 | SELEX | FL |
| D00731.003 | ZNF713 | SELEX | FL |
| D00732.005 | ZNF740 | SELEX | FL |
| D00733.003 | ZNF75A | SELEX | DBD |
| D00734.003 | ZNF784 | SELEX | FL |
| D00735.002 | ZSCAN4 | SELEX | FL |
| D00736.002 | ARID3A | ChIP-seq | K562 |
| D00737.001 | ATF1 | ChIP-seq | K562 |
| D00738.002 | ATF2 | ChIP-seq | H1 |
| D00739.001 | ATF3 | ChIP-seq | H1 |
| D00740.002 | BACH1 | ChIP-seq | K562 |
| D00741.001 | BATF | ChIP-seq | GM12878 |
| D00742.002 | BCL11A | ChIP-seq | H1 |
| D00743.001 | BCL3 | ChIP-seq | GM12878 |
| D00744.001 | BCLAF1 | ChIP-seq | GM12878 |
| D00745.001 | BDP1 | ChIP-seq | K562 |
| D00746.004 | BHLHE40 | ChIP-seq | HepG2 |
| D00747.001 | BRCA1 | ChIP-seq | GM12878 |
| D00748.001 | CBX3 | ChIP-seq | K562 |
| D00749.001 | CCNT2 | ChIP-seq | K562 |
| D00317.009 | CEBPB | ChIP-seq | HeLa |
| D00318.004 | CEBPD | ChIP-seq | HepG2 |
| D00750.001 | CHD1 | ChIP-seq | GM12878 |
| D00751.005 | CHD2 | ChIP-seq | K562 |
| D00752.001 | CTBP2 | ChIP-seq | H1 |
| D00753.001 | CTCFL | ChIP-seq | K562 |
| D00328.018 | CTCF | ChIP-seq | GM12801 |
| D00344.005 | E2F1 | ChIP-seq | HeLa |
| D00347.005 | E2F4 | ChIP-seq | HeLa |
| D00754.003 | E2F6 | ChIP-seq | K562 |
| D00350.005 | EBF1 | ChIP-seq | GM12878 |
| D00351.009 | EGR1 | ChIP-seq | K562 |
| D00356.010 | ELF1 | ChIP-seq | GM12878 |
| D00360.007 | ELK1 | ChIP-seq | GM12878 |
| D00363.004 | ELK4 | ChIP-seq | HeLa |
| D00755.003 | EP300 | ChIP-seq | H1 |
| D00755.005 | EP300 | ChIP-seq | HepG2 |
| D00378.009 | ETS1 | ChIP-seq | GM12878 |
| D00756.007 | EZH2 | ChIP-seq | NHDF |
| D00757.001 | FAM48A | ChIP-seq | HeLa |
| D00758.001 | FOSL1 | ChIP-seq | H1 |
| D00759.001 | FOSL2 | ChIP-seq | HepG2 |
| D00760.003 | FOS | ChIP-seq | K562 |
| D00761.001 | FOXA1 | ChIP-seq | HepG2 |
| D00762.001 | FOXA2 | ChIP-seq | HepG2 |
| D00763.001 | FOXM1 | ChIP-seq | GM12878 |
| D00764.001 | FOXP2 | ChIP-seq | PFSK |
| D00409.004 | GABPA | ChIP-seq | GM12878 |
| D00765.001 | GATA1 | ChIP-seq | K562 |
| D00766.002 | GATA2 | ChIP-seq | K562 |
| D00410.009 | GATA3 | ChIP-seq | SH |
| D00767.001 | GTF2B | ChIP-seq | K562 |
| D00768.001 | GTF2F1 | ChIP-seq | H1 |
| D00769.001 | HDAC1 | ChIP-seq | K562 |
| D00770.002 | HDAC2 | ChIP-seq | HepG2 |
| D00771.001 | HDAC6 | ChIP-seq | K562 |
| D00772.001 | HMGN3 | ChIP-seq | K562 |
| D00446.009 | HNF4A | ChIP-seq | HepG2 |
| D00773.001 | HNF4G | ChIP-seq | HepG2 |
| D00774.001 | IKZF1 | ChIP-seq | GM12878 |
| D00475.006 | IRF3 | ChIP-seq | HeLa |
| D00476.004 | IRF4 | ChIP-seq | GM12878 |
| D00775.001 | JUNB | ChIP-seq | K562 |
| D00776.005 | JUND | ChIP-seq | HepG2 |
| D00777.002 | JUN | ChIP-seq | HUVEC |
| D00778.002 | KAP1 | ChIP-seq | K562 |
| D00779.001 | KDM5A | ChIP-seq | H1 |
| D00780.001 | KDM5B | ChIP-seq | K562 |
| D00501.004 | MAFF | ChIP-seq | HepG2 |
| D00503.014 | MAFK | ChIP-seq | K562 |
| D00504.005 | MAX | ChIP-seq | H1 |
| D00781.003 | MAZ | ChIP-seq | HepG2 |
| D00505.006 | MEF2A | ChIP-seq | K562 |
| D00782.001 | MEF2C | ChIP-seq | GM12878 |
| D00783.001 | MTA3 | ChIP-seq | GM12878 |
| D00784.004 | MXI1 | ChIP-seq | HepG2 |
| D00529.004 | MYBL2 | ChIP-seq | HepG2 |
| D00785.001 | MYC | ChIP-seq | H1 |
| D00786.001 | NANOG | ChIP-seq | H1 |
| D00534.004 | NFATC1 | ChIP-seq | GM12878 |
| D00535.004 | NFE2 | ChIP-seq | K562 |
| D00787.002 | NFIC | ChIP-seq | HepG2 |
| D00788.001 | NFYA | ChIP-seq | GM12878 |
| D00789.003 | NFYB | ChIP-seq | K562 |
| D00552.006 | NR2C2 | ChIP-seq | HepG2 |
| D00790.001 | NR2F2 | ChIP-seq | K562 |
| D00559.006 | NRF1 | ChIP-seq | HeLa |
| D00574.006 | PAX5 | ChIP-seq | GM12891 |
| D00791.001 | PBX3 | ChIP-seq | GM12878 |
| D00792.001 | PHF8 | ChIP-seq | K562 |
| D00793.002 | PML | ChIP-seq | K562 |
| D00794.047 | POLR2A | ChIP-seq | MCF |
| D00589.005 | POU2F2 | ChIP-seq | GM12891 |
| D00795.001 | POU5F1 | ChIP-seq | H1 |
| D00600.004 | PRDM1 | ChIP-seq | HeLa |
| D00796.001 | RAD21 | ChIP-seq | A549 |
| D00797.001 | RBBP5 | ChIP-seq | H1 |
| D00798.001 | RCOR1 | ChIP-seq | HeLa |
| D00799.001 | REST | ChIP-seq | GM12878 |
| D00619.007 | RFX5 | ChIP-seq | H1 |
| D00800.001 | RPC155 | ChIP-seq | HeLa |
| D00624.009 | RUNX3 | ChIP-seq | GM12878 |
| D00626.009 | RXRA | ChIP-seq | GM12878 |
| D00801.001 | SAP30 | ChIP-seq | K562 |
| D00802.001 | SETDB1 | ChIP-seq | K562 |
| D00803.002 | SIN3AK20 | ChIP-seq | HepG2 |
| D00804.002 | SIN3A | ChIP-seq | H1 |
| D00805.001 | SIRT6 | ChIP-seq | K562 |
| D00806.003 | SIX5 | ChIP-seq | K562 |
| D00807.001 | SMARCB1 | ChIP-seq | HeLa |
| D00808.004 | SMC3 | ChIP-seq | K562 |
| D00650.007 | SP1 | ChIP-seq | GM12878 |
| D00809.002 | SP2 | ChIP-seq | K562 |
| D00652.004 | SP4 | ChIP-seq | H1 |
| D00655.006 | SPI1 | ChIP-seq | K562 |
| D00660.007 | SRF | ChIP-seq | H1 |
| D00810.001 | STAT3 | ChIP-seq | HeLa |
| D00811.001 | STAT5A | ChIP-seq | GM12878 |
| D00812.001 | SUZ12 | ChIP-seq | H1 |
| D00813.009 | TAF1 | ChIP-seq | SK |
| D00814.001 | TAF7 | ChIP-seq | H1 |
| D00815.001 | TAL1 | ChIP-seq | K562 |
| D00816.001 | TBL1XR1 | ChIP-seq | GM12878 |
| D00817.001 | TBP | ChIP-seq | GM12878 |
| D00818.003 | TCF12 | ChIP-seq | HepG2 |
| D00819.002 | TCF7L2 | ChIP-seq | HEK293 |
| D00679.004 | TEAD4 | ChIP-seq | H1 |
| D00681.004 | TFAP2A | ChIP-seq | HeLa |
| D00683.007 | TFAP2C | ChIP-seq | HeLa |
| D00820.001 | THAP1 | ChIP-seq | K562 |
| D00821.001 | TRIM28 | ChIP-seq | K562 |
| D00822.001 | UBTF | ChIP-seq | K562 |
| D00700.006 | USF1 | ChIP-seq | HepG2 |
| D00823.002 | USF2 | ChIP-seq | H1 |
| D00824.001 | WRNIP1 | ChIP-seq | GM12878 |
| D00710.007 | YY1 | ChIP-seq | H1 |
| D00825.001 | ZBTB33 | ChIP-seq | GM12878 |
| D00714.004 | ZBTB7A | ChIP-seq | HepG2 |
| D00826.001 | ZEB1 | ChIP-seq | GM12878 |
| D00723.006 | ZNF143 | ChIP-seq | HeLa |
| D00827.001 | ZNF217 | ChIP-seq | MCF |
| D00828.002 | ZNF263 | ChIP-seq | K562 |
| D00829.002 | ZNF274 | ChIP-seq | K562 |
