# Supplementary material for: GRAM: A GeneRAlized Model to predict the molecular effect of a non-coding variant in a cell-type specific manner
Source: PLoS Genet. 2019 Aug 30;15(8):e1007860. doi: 10.1371/journal.pgen.1007860 (PMC6742416; doi:10.1371/journal.pgen.1007860)
Supplement: S3 Table — (DOCX) [file pgen.1007860.s003.docx]

**S3 Table** Primers for 14 regions cloning in K562

| **Element Number** | **Forward Primer** | **Reverse Primer** |
| --- | --- | --- |
| 1 | GGGGACAACTTTGTACAAAAAAGTTGGCACCGGTACACGAAGGCTGGG | GGGGACAACTTTGTACAAGAAAGTTGGCAACTGCTGTAGCCCACCA |
| 2 | GGGGACAACTTTGTACAAAAAAGTTGGCACCGGCAGTTGGAAAGGGC | GGGGACAACTTTGTACAAGAAAGTTGGCAAGTCCTCAGGAACCGGC |
| 3 | GGGGACAACTTTGTACAAAAAAGTTGGCACCCGTGATCAACCAAAATCACCTCA | GGGGACAACTTTGTACAAGAAAGTTGGCAATCCTCTGCTCTACGGAATGGA |
| 4 | GGGGACAACTTTGTACAAAAAAGTTGGCACCTATCCGGAAGGGGTGGAACC | GGGGACAACTTTGTACAAGAAAGTTGGCAACCCCAGGTCACTCAGACCAC |
| 5 | GGGGACAACTTTGTACAAAAAAGTTGGCACCTAAGCCCAGTCCTCAGC | GGGGACAACTTTGTACAAGAAAGTTGGCAAAAGGAAGACTAATTGGGTCCC |
| 6 | GGGGACAACTTTGTACAAAAAAGTTGGCACCAGAACTAGCAGCGGAGAC | GGGGACAACTTTGTACAAGAAAGTTGGCAATCCTTCTTGCATGGTCGG |
| 7 | GGGGACAACTTTGTACAAAAAAGTTGGCACCTCCCCTGCTTTTCGATCTCTC | GGGGACAACTTTGTACAAGAAAGTTGGCAACTTTAGCCGCCCCTTTCCA |
| 8 | GGGGACAACTTTGTACAAAAAAGTTGGCACCCCTTTTCTAATCACCATTTCCGATA | GGGGACAACTTTGTACAAGAAAGTTGGCAATAACAGGGGCAATGCACTTT |
| 9 | GGGGACAACTTTGTACAAAAAAGTTGGCACCTGTTTTGCTTTTAACCTGATGAT | GGGGACAACTTTGTACAAGAAAGTTGGCAAAGGAGGTGGGGTGGCT |
| 10 | GGGGACAACTTTGTACAAAAAAGTTGGCACCCTTTCACGCAAAACCTGCTCA | GGGGACAACTTTGTACAAGAAAGTTGGCAAAACCTTTTTCCTTGAACCTTAGAC |
| 11 | GGGGACAACTTTGTACAAAAAAGTTGGCACCGGTCTTGACGCTGGCC | GGGGACAACTTTGTACAAGAAAGTTGGCAATGCTGGGAGAAACCGAT |
| 12 | GGGGACAACTTTGTACAAAAAAGTTGGCACCGGCTTCACTAACCTTAAATTCTAAA | GGGGACAACTTTGTACAAGAAAGTTGGCAATGCCCTTAAACAAGATGGC |
| 13 | GGGGACAACTTTGTACAAAAAAGTTGGCACCGTTCCCCTTCTGTCTCAGG | GGGGACAACTTTGTACAAGAAAGTTGGCAATTCATTCAGGGGCTCCC |
| 14 | GGGGACAACTTTGTACAAAAAAGTTGGCACCGACCCCACCCCTTCCC | GGGGACAACTTTGTACAAGAAAGTTGGCAATTTTGGCAGGAGCAGGA |
