# Supplementary figures and images for: GRAM: A GeneRAlized Model to predict the molecular effect of a non-coding variant in a cell-type specific manner
Source: PLoS Genet. 2019 Aug 30;15(8):e1007860. doi: 10.1371/journal.pgen.1007860 (PMC6742416; doi:10.1371/journal.pgen.1007860)

**S1 Fig** Distribution of conservation scores among different annotation categories

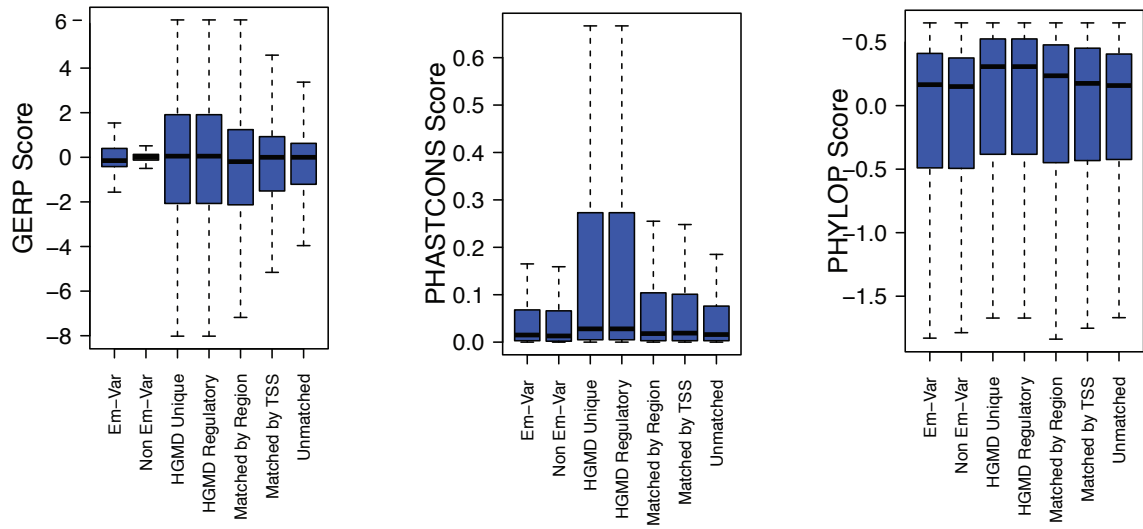

Supplement: S1 Fig — (PDF) [file pgen.1007860.s005.pdf]

**S2 Fig** Availability of different data types in ENCODE

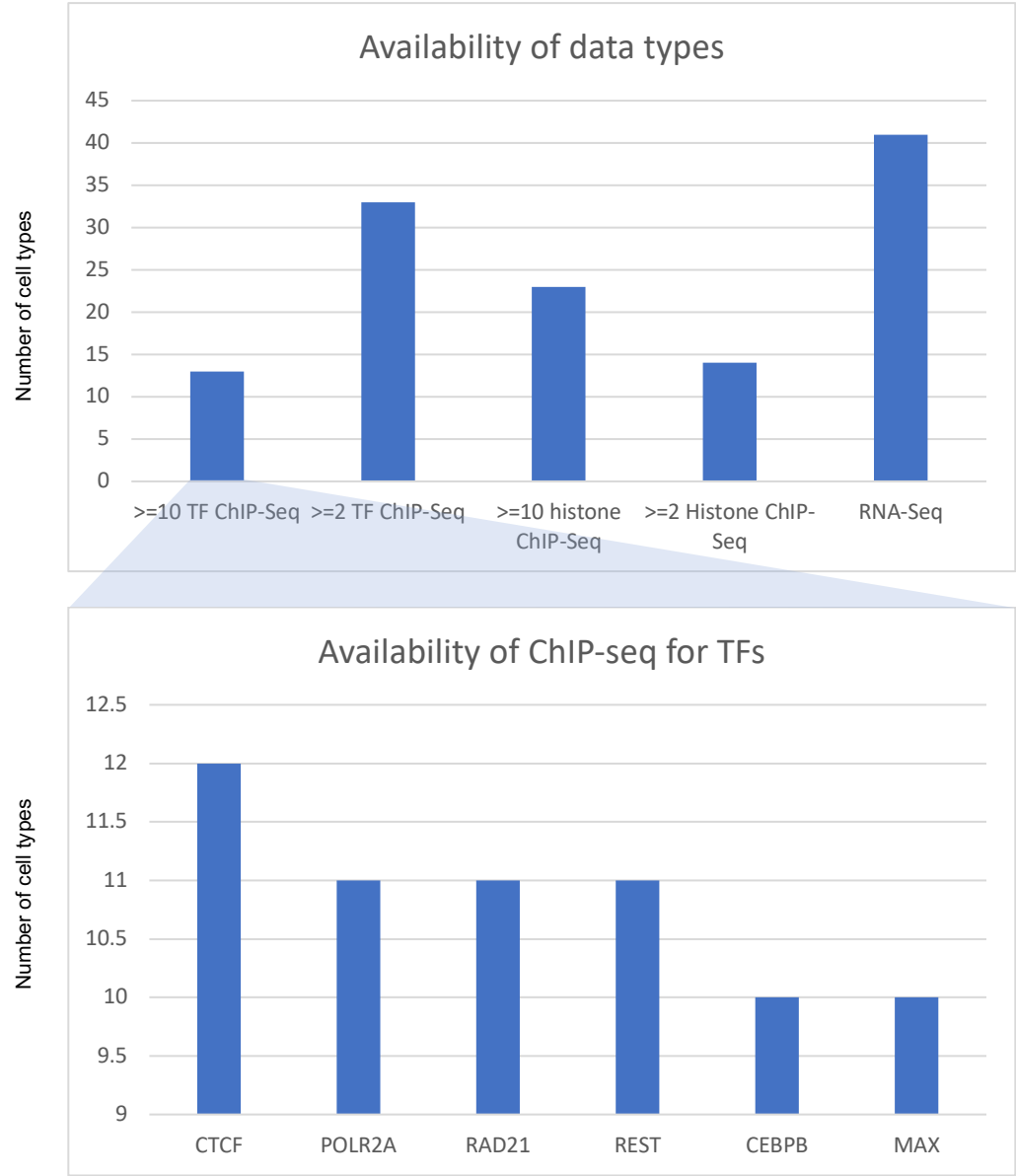

Supplement: S2 Fig — (PDF) [file pgen.1007860.s006.pdf]

**S3 Fig** PRC curve for regulatory activity prediction

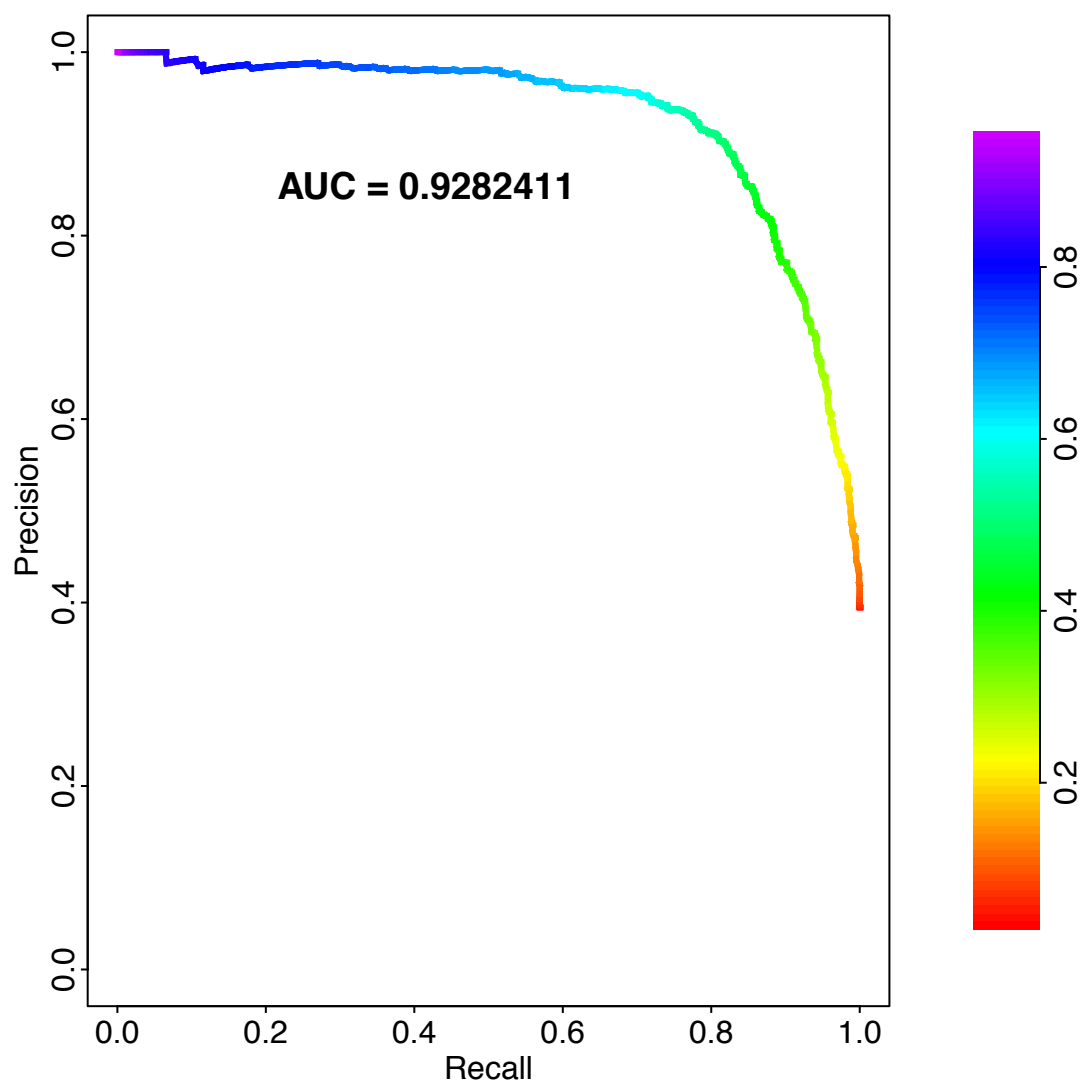

Supplement: S3 Fig — (PDF) [file pgen.1007860.s007.pdf]

**S4 Fig** Principal component analysis using Vodds for three cell lines: GM12878, GM19239 and HepG2

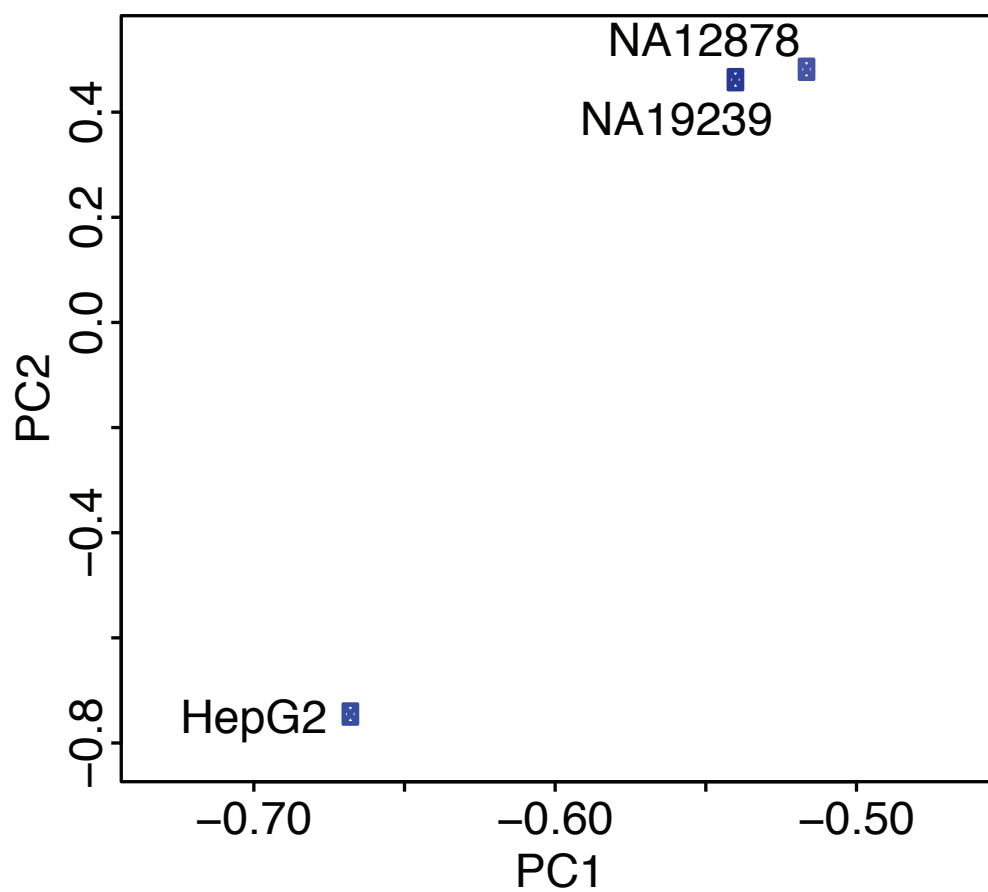

Supplement: S4 Fig — (PDF) [file pgen.1007860.s008.pdf]

**S6 Fig** The prediction of cell type modifier score using TF binding profiles.

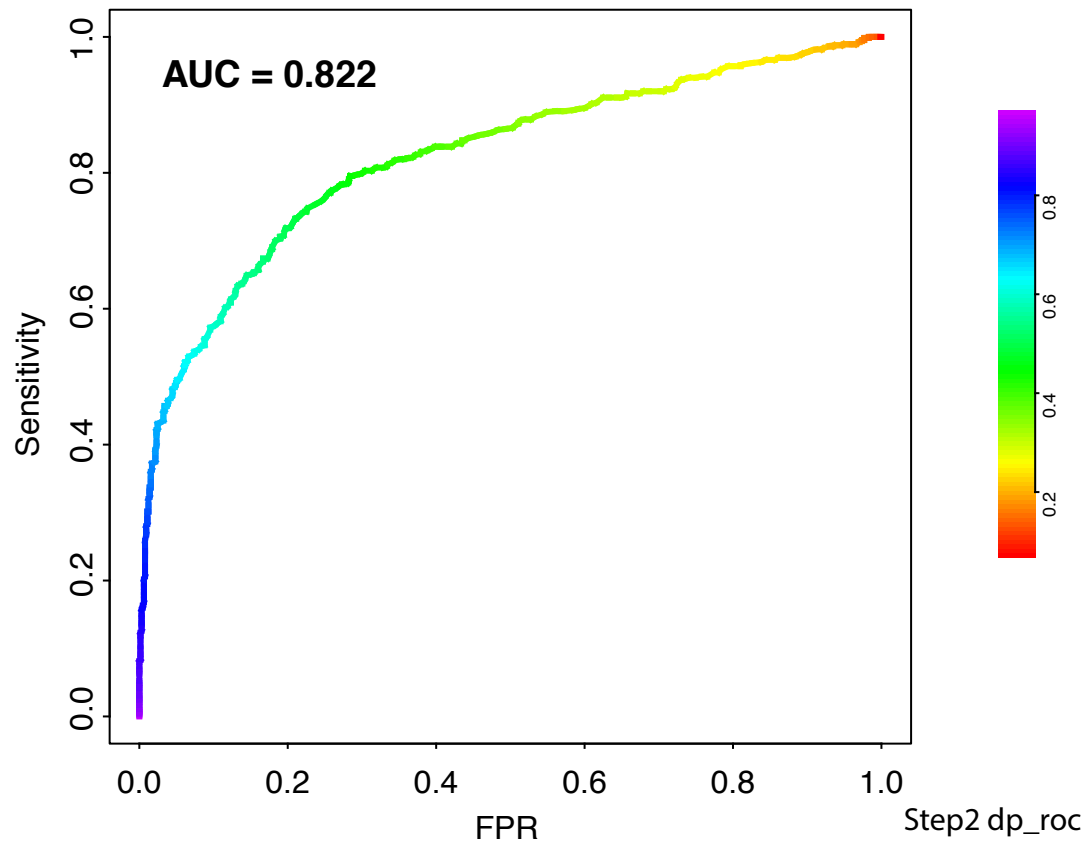

Supplement: S6 Fig — (PDF) [file pgen.1007860.s010.pdf]

**S8 Fig** The training and cross-validation scheme.

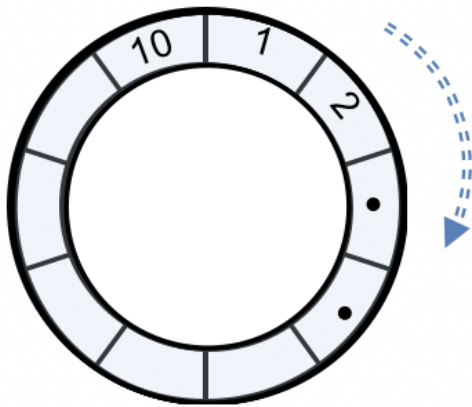

Supplement: S8 Fig — (PDF) [file pgen.1007860.s012.pdf]

**S9 Fig** The GRAMMAR pipeline.

GRAMMAR pipeline

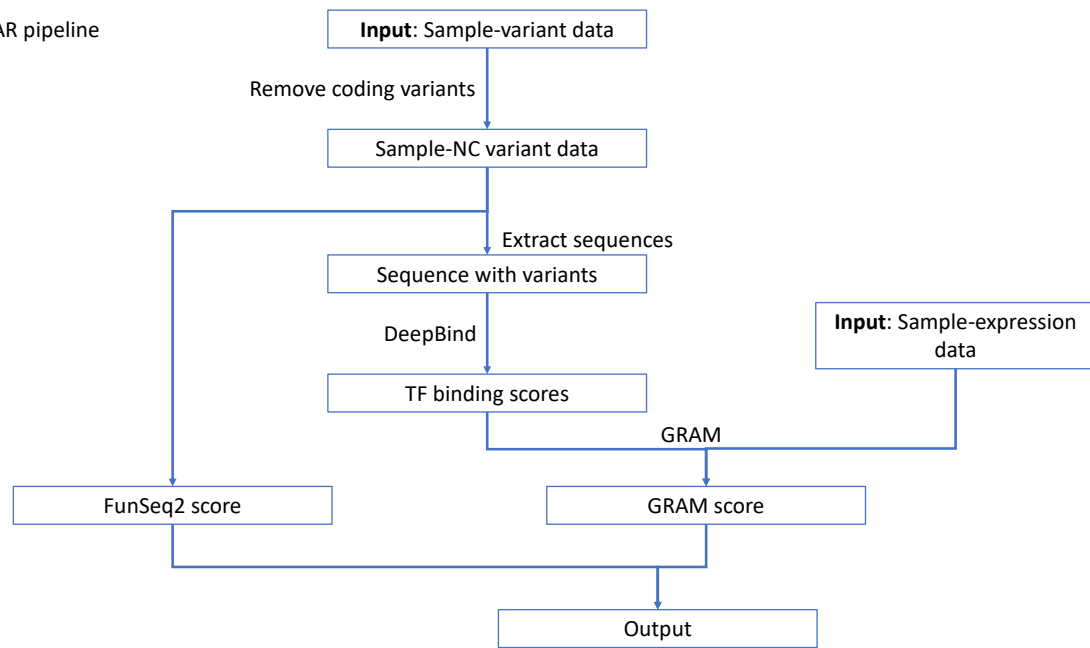

Supplement: S9 Fig — (PDF) [file pgen.1007860.s013.pdf]
