# Supplementary material for: GRAM: A GeneRAlized Model to predict the molecular effect of a non-coding variant in a cell-type specific manner
Source: PLoS Genet. 2019 Aug 30;15(8):e1007860. doi: 10.1371/journal.pgen.1007860 (PMC6742416; doi:10.1371/journal.pgen.1007860)

S5 Fig Distribution of Vodds score for GM12878. The high and low variable cell specificity class are defined by the top and bottom quantile.

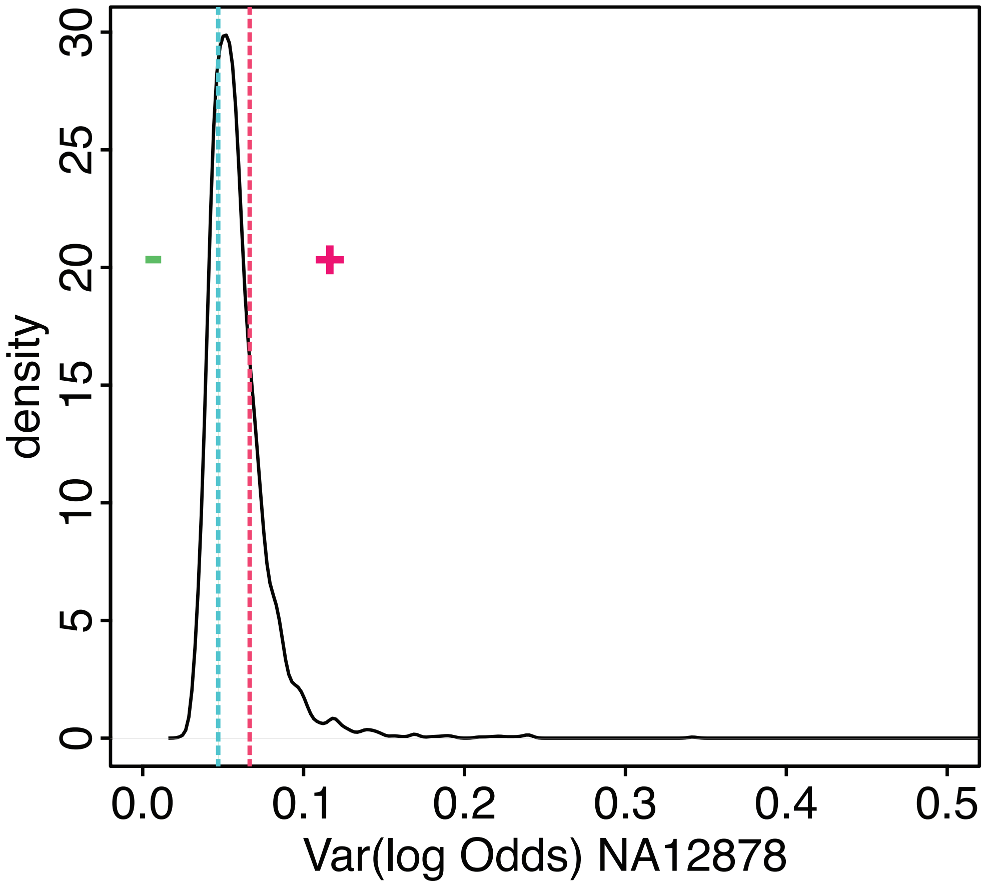

Supplement: S5 Fig — The high and low variable cell specificity class are defined by the top and bottom quantile. (PDF) [file pgen.1007860.s009.pdf]
