# Supplementary material for: GRAM: A GeneRAlized Model to predict the molecular effect of a non-coding variant in a cell-type specific manner
Source: PLoS Genet. 2019 Aug 30;15(8):e1007860. doi: 10.1371/journal.pgen.1007860 (PMC6742416; doi:10.1371/journal.pgen.1007860)

S7 Fig Comparison of average GRAM scores with high land low posterior probability reported by Dadaev et al.

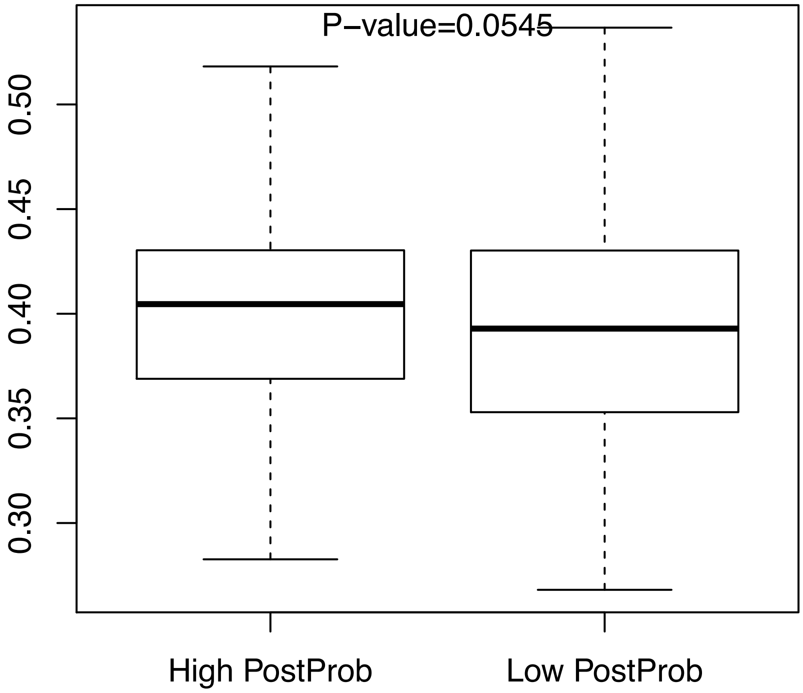

Supplement: S7 Fig — (PDF) [file pgen.1007860.s011.pdf]
